# Supplementary material for: An ancient role for CYP73 monooxygenases in phenylpropanoid biosynthesis and embryophyte development
Source: EMBO J. 2024 Aug 1;43(18):13. doi: 10.1038/s44318-024-00181-7 (PMC11405693; doi:10.1038/s44318-024-00181-7)
Supplement: Supplementary file 1 — Appendix [file 44318_2024_181_MOESM1_ESM.pdf]

An ancient role for CYP73 monooxygenases in phenylpropanoid biosynthesis and embryophyte development

Appendix

p. 2 | Appendix Figure S1. *In vitro* C4H assay with *Klebsormidium nitens* kfl00038_0230 recombinant protein.

p. 3 | Appendix Figure S2. Cytochromes P450 CO difference spectra of wild-type and R>A mutated CYP73 proteins.

p. 4 | Appendix Figure S3. Western-blot analysis of recombinant PpPpCYP73A-3xHA proteins.

p. 5 | Appendix Figure S4. Molecular characterization of *Physcomitrium patens* ∆*CYP73A* mutants.

p. 6 | Appendix Figure S5. Macroscopic phenotypes of *Physcomitrium patens* ∆*CYP73A* mutants.

p. 7 | Appendix Figure S6. Expression analysis of *PpCYP73A48* and *PpCYP73A49* genes in mutant backgrounds.

p. 8 | Appendix Figure S7. Chemical complementation of ∆*PpCYP73A48/CYP73A49* gametophore stunted growth with *p*-coumaric acid.

p. 9 | Appendix Figure S8. Search for *cis*-cinnamic acid in C4H-impaired plants.

p. 10 | Appendix Figure S9. Molecular characterization of *Mpcyp73a1* CRISPR mutants.

p. 11 | Appendix Table S1. Search for CYP73 homologs in Viridiplantae proteomes by reciprocal best hits (RBH).

p. 12 | Appendix Table S2. Quantification of free hydroxycinnamic acids in crude extracts of *M. polymorpha* and *A. agrestis* after PA treatment.

p. 13 | Appendix Table S3. List of primers and synthesized sequences used in the study.

p. 15 | Appendix Table S4. List of multiple reaction monitoring (MRM) methods used for targeted analysis by UHPLC-MS/MS.

p. 16 | Appendix Table S5. List of quantification masses and molar response factors used for quantitative analysis of cuticular monomers by GC-TOFMS.

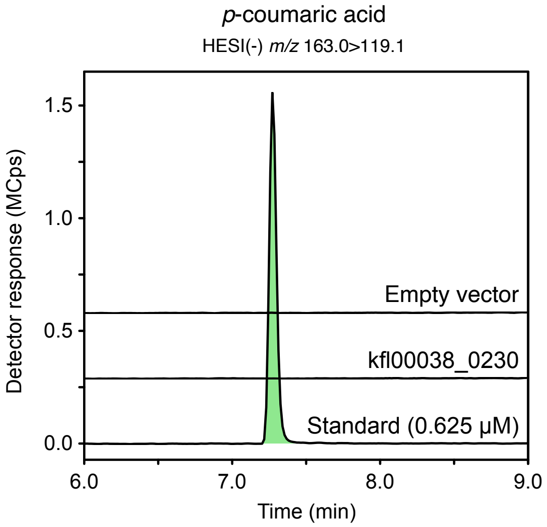

Appendix Figure S1. *In vitro* C4H assay with *Klebsormidium nitens* kfl00038_0230 recombinant protein. Representative UHPLC-MS/MS chromatograms showing the absence of *p*-coumaric acid production from *t*-cinnamic acid (C4H activity) in *in vitro* assay using microsomes from yeasts transformed with the pYeDP60:kfl00038_0230 plasmid. Negative control assays were performed with microsomes from yeasts transformed with an empty vector.

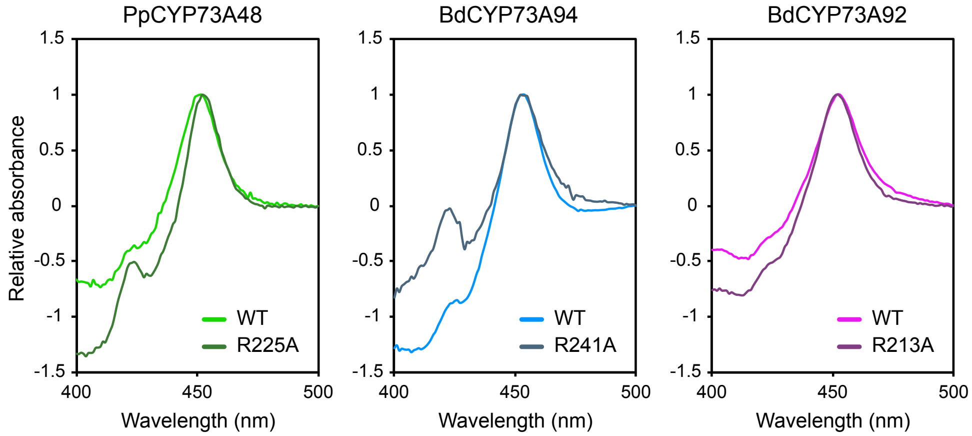

Appendix Figure S2. Cytochromes P450 CO difference spectra of wild-type and R>A mutated CYP73 proteins. Properly folded CYPs exhibit a maximum absorbance at 450 nm when bound to carbon monoxide. CO difference spectra were determined from 20-fold dilution of microsomal preparations. Each absorbance spectrum was normalized according to its maximum (set to 1).

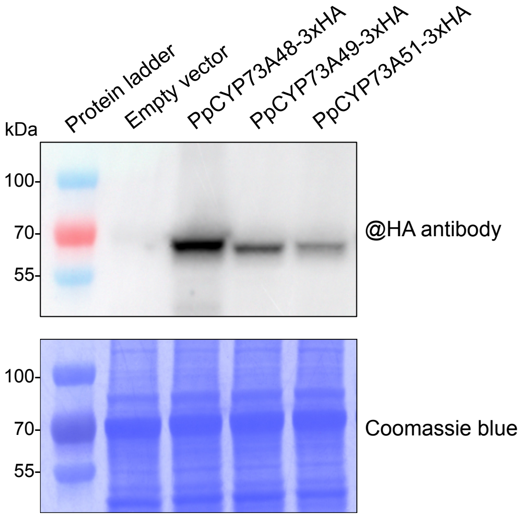

Appendix Figure S3. Western-blot analysis of recombinant PpPpCYP73A-3xHA proteins. Microsomes were 10-fold diluted in denaturation buffer (200 mM Tris pH 6.8, 8% SDS, 40% glycerol, 0.1% bromophenol blue and 100 mM DTT) prior to denaturation at 65°C for 5 min. Ten micrograms total protein were loaded on each lane.

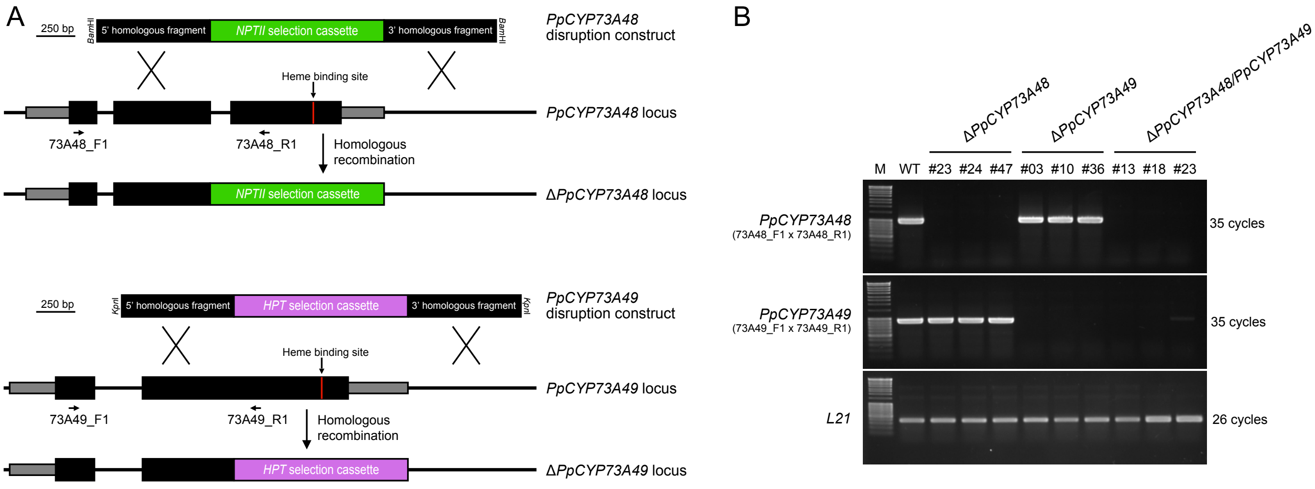

Appendix Figure S4. Molecular characterization of *Physcomitrium patens* Δ*CYP73A* mutants. (A) Homologous recombination-mediated strategy for *PpCYP73A48* and *PpCYP73A49* gene disruption. Genomic fragments encompassing the critical heme-binding site were excised with simultaneous insertion of the *NPTII* (*PpCYP73A48*) or *HPT* (*PpCYP73A49*) selection cassette conferring resistance to geneticin and hygromycin B, respectively. (B) RT-PCR analysis of Δ*PpCYP73A48,* Δ*PpCYP73A49* and Δ*PpCYP73A48/PpCYP73A49* mutant lines showing the absence of corresponding transcripts. The primer hybridization sites for RT-PCR are indicated in panel A. RT-PCR analysis of *L21* transcripts was used as amplification control. M, MassRuler™ DNA Ladder (ThermoFisher Scientific).

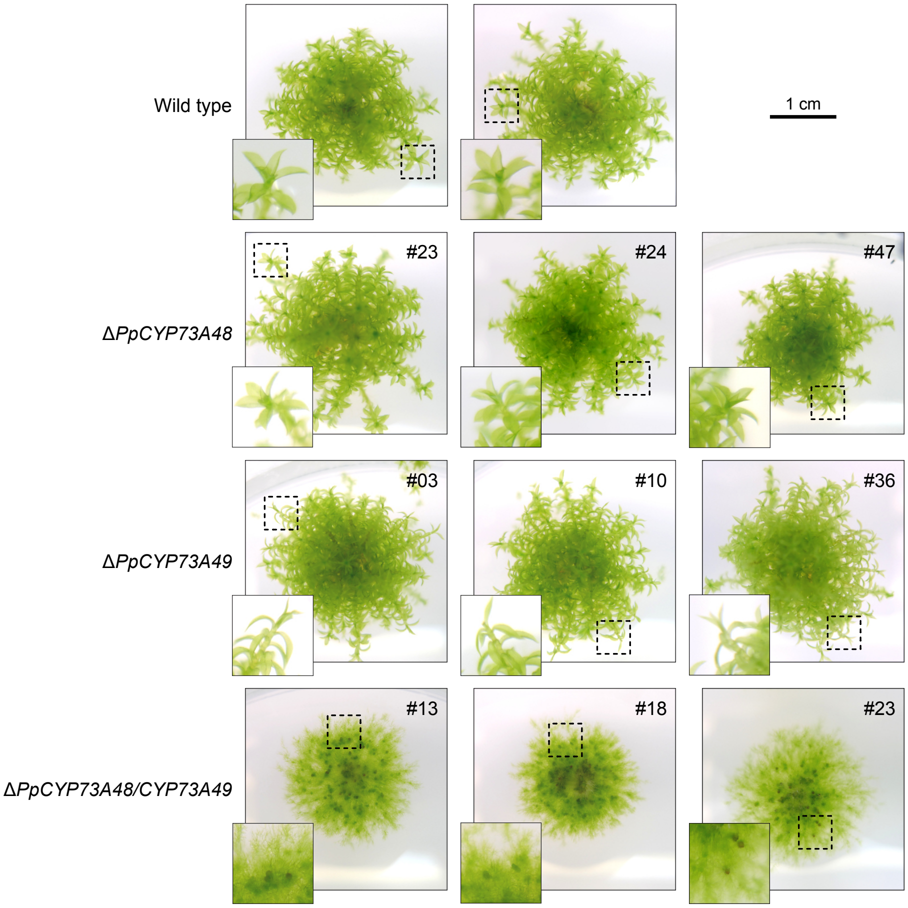

Appendix Figure S5. Macroscopic phenotypes of *Physcomitrium patens* Δ*CYP73A* mutants. Pictures of two-month-old *P. patens* colonies highlighting the consistent phenotypes observed across three independent lines for each genotype. For each colony, a 2.4x magnification of boxed area is shown.

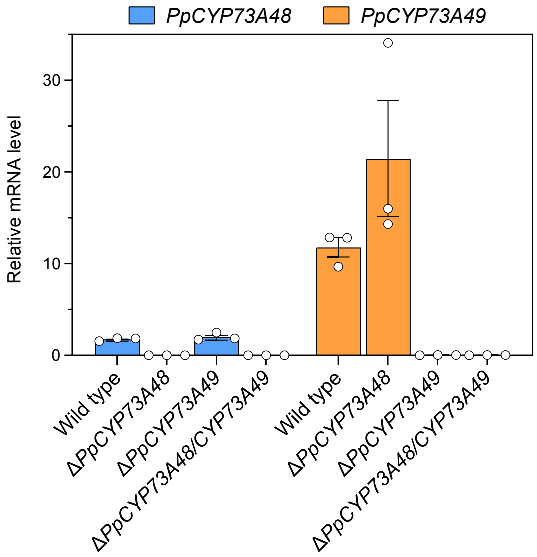

Appendix Figure S6. Expression analysis of *PpCYP73A48* and *PpCYP73A49* genes in mutant backgrounds. Relative mRNA levels were determined by RT-qPCR in wild type, Δ*PpCYP73A48,* Δ*PpCYP73A49* and Δ*PpCYP73A48/CYP73A49* mutant lines. Results are the mean ± SEM of three independent WT biological replicates and three independent mutant lines.

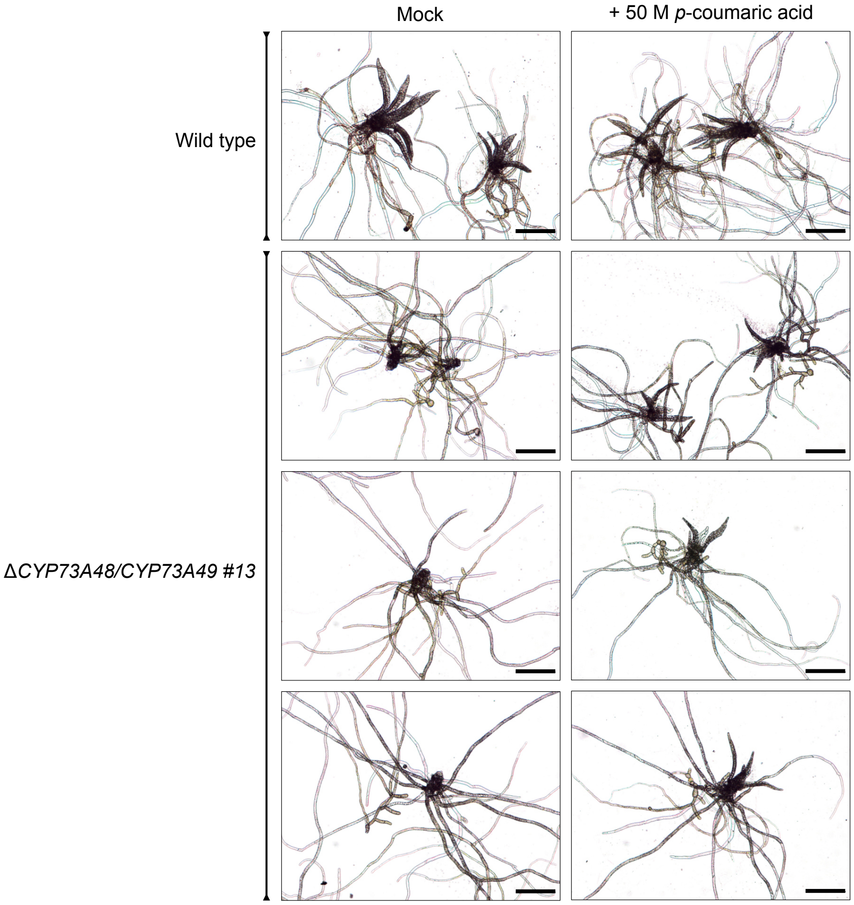

Appendix Figure S7. Chemical complementation of Δ*PpCYP73A48/CYP73A49* gametophore stunted growth with *p*-coumaric acid. Protoplasts from wild type and Δ*PpCYP73A48/CYP73A49* #13 mutant line were embedded in low-melting point agarose and regenerated in the presence of 50 µM *p*-coumaric acid or a corresponding mock medium (0.1% ethanol). Pictures were taken five weeks after start of regeneration and illustrate the positive effect of *p*-coumaric acid in rescuing the stunted growth of Δ*PpCYP73A48/CYP73A49* #13 gametophores. No significant effects of *p*-coumaric acid were observed in the wild type. Scale bars, 0.25 mm.

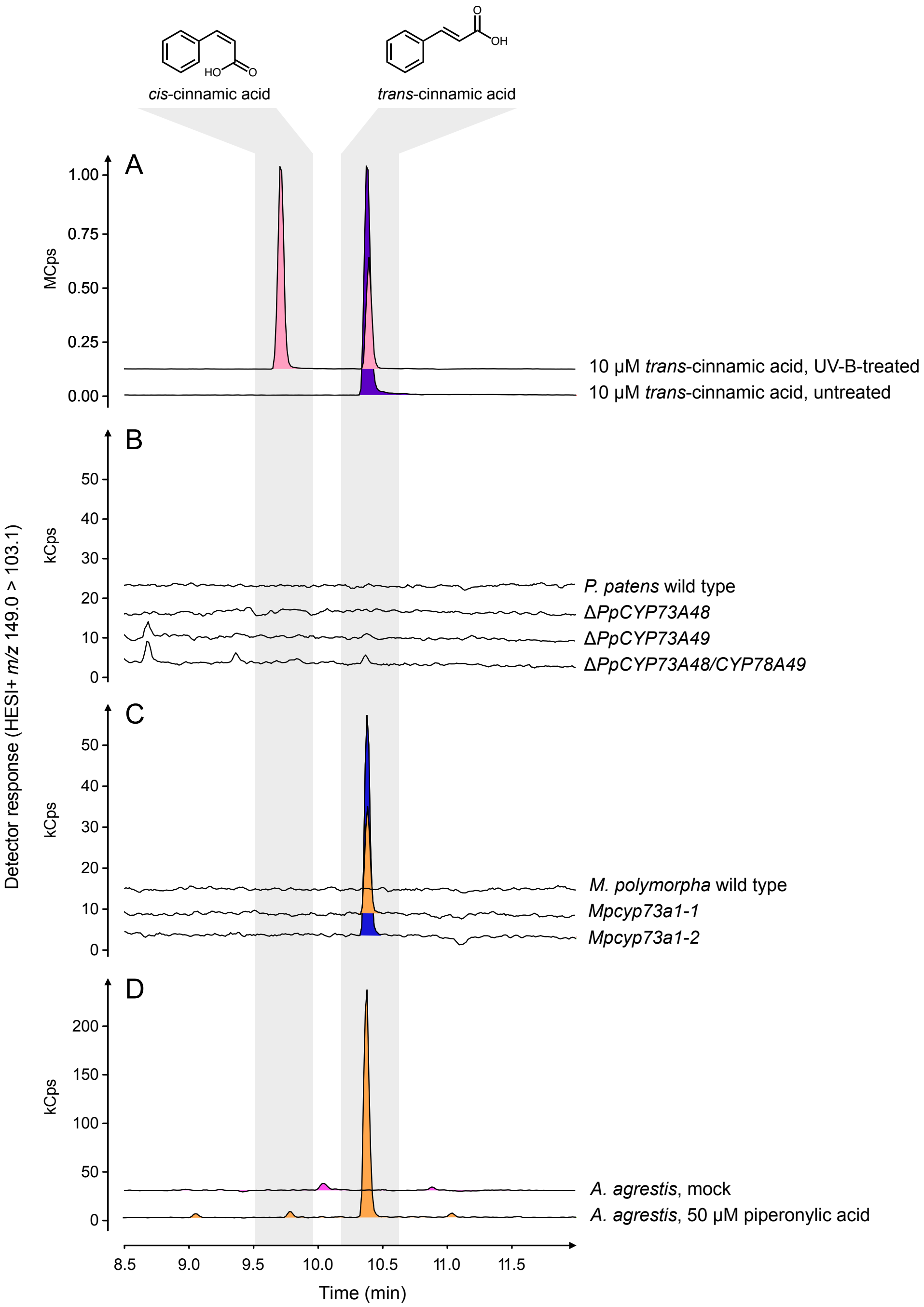

Appendix Figure S8. Search for *cis*-cinnamic acid in C4H-impaired plants. (A) A 10 µM *trans*-cinnamic acid solution was treated for 15 min with UV-B light provided by four UVB Broadband TL 40W/12 RS SLV tubes (Phillips), resulting in the appearance of the stereoisomer *cis*-cinnamic acid. Both isomers were then searched by UHPLC-MS/MS in crude extracts of (B) *P. patens* wild type and Δ*CYP73A* mutants (see Fig. 4), (C) *M. polymorpha* wild type and *Mpcyp73a1* CRISPR mutants (see Fig. 5), and in (D) *A. agrestis* treated with 50 µM piperonylic acid or corresponding mock treatment (see Fig. 6). Shown are representative UHPLC-MS/MS chromatograms.

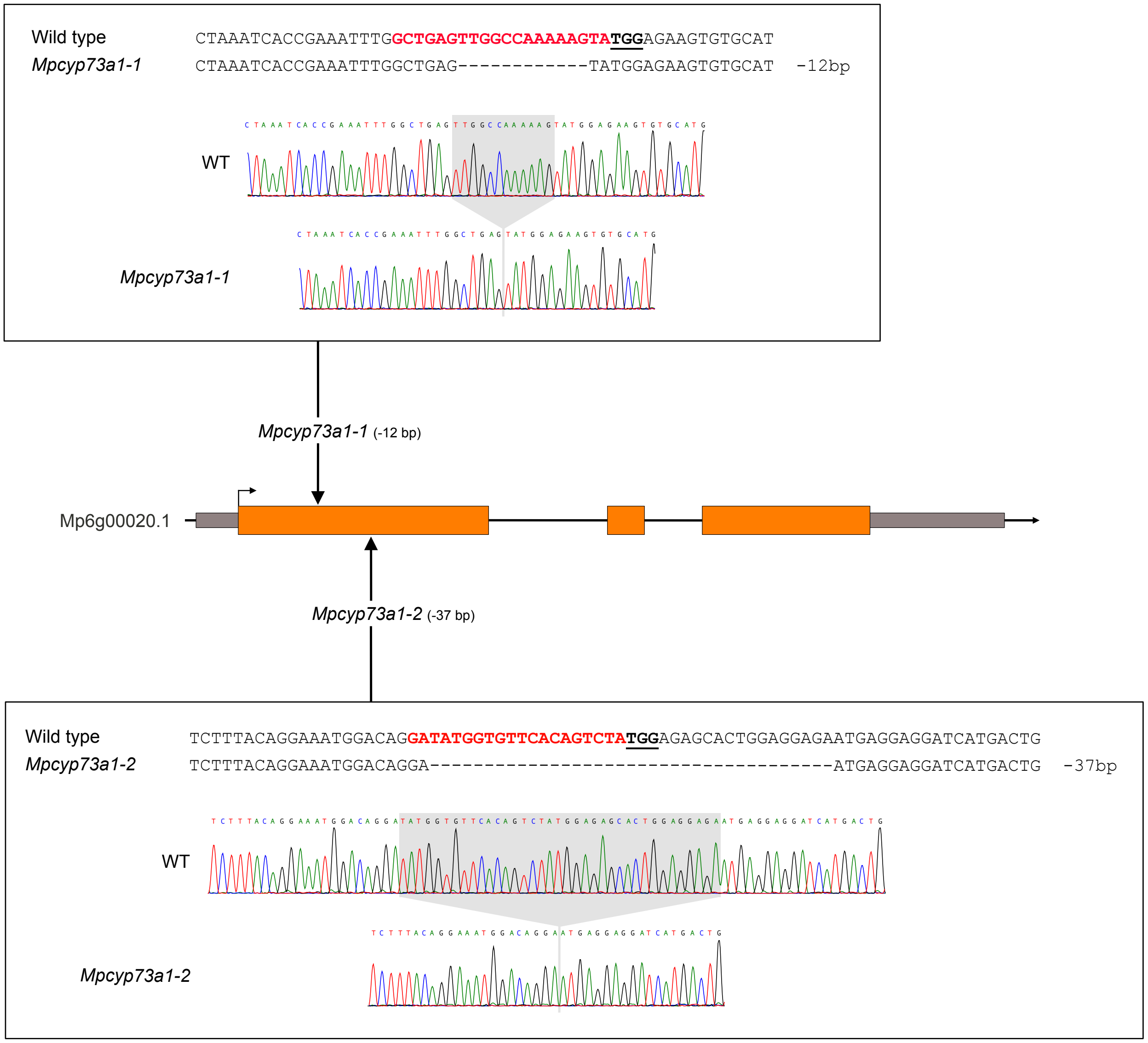

Appendix Figure S9. Molecular characterization of *Mpcyp73a1* CRISPR mutants. Two independent *Mpcyp73a1* CRISPR mutant lines were isolated and characterized by Sanger sequencing. The protospacer sequences, their corresponding locations, and the sequencing results are shown for both mutant lines. *Mpcyp73a1-1* exhibits a deletion of 12 nucleotides, while *Mpcyp73a1-2* displays a deletion of 37 nucleotides.

Appendix Table S1. Search for CYP73 homologs in Viridiplantae proteomes by reciprocal best hits (RBH). Forward BLASTp search across 20 Viridiplantae species was performed using *A. thaliana* CYP73A5 protein sequence as query. For each species, the top hit based on the bit-score was used to perform a reverse BLASTp search against the *Arabidopsis thaliana* proteome.

Plant species	Forward BLASTp				Reverse BLASTp	
	Best hit	Bit-score	e-value	identity	Best hit	Bit-score
Arabidopsis thaliana	AT2G30490	1037	0	100%	AT2G30490 | CYP73A5	1037
Brachypodium distachyon	Bradi2g53470	862	0	80%	AT2G30490 | CYP73A5	839
Amborella trichopoda	scaffold00077.91	873	0	83%	AT2G30490 | CYP73A5	853
Picea abies	MA_118702g0010	814	0	79%	AT2G30490 | CYP73A5	813
Thuja plicata	Thupl.29378841s0014	810	0	79%	AT2G30490 | CYP73A5	814
Salvinia cucullata	s0039.g012203	755	0	76%	AT2G30490 | CYP73A5	749
Ceratopteris richardii	Ceric.38G063100	757	0	72%	AT2G30490 | CYP73A5	738
Diphasiastrum complanatum	Dicom.04G054900	782	0	76%	AT2G30490 | CYP73A5	764
Selaginella moellendorffii	175973	714	0	71%	AT2G30490 | CYP73A5	720
Physcomitrium patens	Pp3c4_21680	767	0	73%	AT2G30490 | CYP73A5	747
Marchantia polymorpha	Mp6g00020	706	0	71%	AT2G30490 | CYP73A5	718
Anthoceros agrestis	228.5028.1	748	0	72%	AT2G30490 | CYP73A5	743
Penium margaritaceum	pm004896g0030	116	3e-26	26%	AT2G26170 | CYP711A1	208
Zygnema circumcaritanum	Zci_08574.1	162	4e-42	28%	AT2G40890 | CYP98A3	310
Mesotaenium endlicherianum	ME000292S04845	115	8e-26	27%	AT1G31800 | CYP97A3	695
Spirogloea muscicola	SM000094S24703	111	2e-24	24%	AT1G31800 | CYP97A3	709
Chara braunii	CHBRA18g00060	163	2e-41	35%	AT2G45560 | CYP76C1	187
Klebsormidium nitens	kfl00038_0230	235	1e-69	31%	AT2G40890 | CYP98A3	260
Chlamydomonas reinhardtii	Cre02.g142266	119	4e-27	25%	AT1G31800 | CYP97A3	590
Ostreococcus lucimarinus	gwEuk.21.62.1	114	7e-26	25%	AT1G31800 | CYP97A3	565

Appendix Table S2. Quantification of free hydroxycinnamic acids in crude extracts of *M. polymorpha* and *A. agrestis* after PA treatment. HCAA levels are expressed as nmoles/g dry weight. Results are the mean ± SEM of three independent biological replicates for each condition. Mock versus PA unpaired *t* test adjusted P-value: **P*<0.05; ***P*<0.01; ****P*<0.001. nd, not detected.

	M. polymorpha		***A. agrestis***	
	Mock	PA	Mock	PA
t-cinnamic acid	n.d	961.2 ±69.5	n.d	244.2 ±14.8
p-coumaric acid	39.4 ±2.1	***7.9 ±0.9	8089.5 ±406.1	***59.6 ±9.1
caffeic acid	0.6 ±0.1	n.d	347.4 ±19.1	**184.2 ±26.1
ferulic acid	57.9 ±2.7	***2.9 ±0.3	35.3 ±1.2	***0.8 ±0.4

Appendix Table S3. List of primers and synthesized sequences used in the study.

ID	Name	Sequence (5'>3')
**Gene expression analysis by RT-qPCR**		
SK0561	MpACT7_qF	AGGCATCTGGTATCCACGAG
SK0562	MpACT7_qR	ACATGGTCGTTCCTCCAGAC
SK0563	MpEF1a_qF	CCGAGATCCTGACCAAGG
SK0564	MpEF1a_qR	GAGGTGGGTACTCAGCGAAG
SK0569	MpCYP73A1_qF	TATCCTCGGTCGTGGACACT
SK0570	MpCYP73A1_qR	AGAAGGGGAATGGCCATGTG
SK0565	MpCYP73A2_qF	GCGCTCAAGGACAAGAGACT
SK0566	MpCYP73A2_qR	TGAAGTCGATGGCCACCTTC
SK0567	MpCYP73A3_qF	ATGAGCTGGACACGGTTCTG
SK0568	MpCYP73A3_qR	TTCACCACCGCCGTCAAATA
**Characterization of *P. patens* mutants by RT-PCR**		
HR635	L21_sqF	GGTTGGTCATGGGTTGCG
HR636	L21_sqR	GAGGTCAACTGTCTCGCC
HR629	73A48_F1	CGTGCAAGGATTATGCGTGG
HR630	73A48_R1	TCAGGCTCGCTCACCAAATT
HR627	73A49_F1	TAATGCAGCGGTGTCGAGTT
HR628	73A49_R1	GCCGCGACGTTAATGTTCTC
**Genotyping of *cyp73a5-1* mutant line**		
HR216	GABI_o8409	ATATTGACCATCATACTCATTGC
HR158	GK_753B06_LP	TCAGCAGCTTCTTCTGCTTTC
HR159	GK_753B06_RP	CCTTATGCAAGCAGAGACGTC
**Protospacers for CRISPR/Cas9 (Golgen Gate cloning)**		
HR1279	MpCYP73A1_gRNA1_BsaI_F	ctcgGCTGAGTTGGCCAAAAAGTA
HR1280	MpCYP73A1_gRNA1_BsaI_R	aaacTACTTTTTGGCCAACTCAGC
HR1281	MpCYP73A1_gRNA2_BsaI_F	ctcgGATATGGTGTTCACAGTCTA
HR1282	MpCYP73A1_gRNA2_BsaI_R	aaacTAGACTGTGAACACCATATC
**Genotyping of CRISPR/Cas9 lines (PCR & Sanger sequencing)**		
HR1283	MpCYP73A1_gRNA1/2_screen_F	GCTTCAAGCAGCAGCATGTT
HR1284	MpCYP73A1_gRNA1/2_screen_R	ACATCATGAGCTGCAGCCTT
**CDS cloning (Gateway® cloning)**		
HR438	PpCYP73A48_AttB1	ggggacaagtttgtacaaaaaagcaggcttcATGGCGGGCACAATAACGAT
HR439	PpCYP73A48_AttB2	ggggaccactttgtacaagaaagctgggtcCTAGACGGCAAGAGGTCTGG
HR436	PpCYP73A49_AttB1	ggggacaagtttgtacaaaaaagcaggcttcATGGGAGCAGGATGGAAGGA
HR437	PpCYP73A49_AttB2	ggggaccactttgtacaagaaagctgggtcTTAAGCGATGGGTTTGCAGAC
HR440	PpCYP73A51_AttB1	ggggacaagtttgtacaaaaaagcaggcttcATGGGGAAGCCAATGGGC
HR441	PpCYP73A51_AttB2	ggggaccactttgtacaagaaagctgggtcCTAGGCTCTCGGCCTTGTG
HR1293	MpCYP73A1_ AttB1	ggggacaagtttgtacaaaaaagcaggcttcATGTGGGGTGAGAGAGCAGC
HR1294	MpCYP73A1_ AttB2	ggggaccactttgtacaagaaagctgggtcTCAATCAGCCCTCGGTTTAACA
**Site-directed mutagenesis**		
HR642	PpCYP73A48_R225A_F	GAGCCAACCGACTGGCCTCCCCGTTAAGTG
HR643	PpCYP73A48_R225A_R	CACTTAACGGGGAGGCCAGTCGGTTGGCTC
HR646	CYP73A94opti_R241A_F	GTGCTAGACGGCTAGCTTCGGAATTAAATCTGGTAGCTTGGA
HR647	CYP73A94opti_R241A_R	TCCAAGCTACCAGATTTAATTCCGAAGCTAGCCGTCTAGCAC
HR549	CYP73A92opti_R213A_F	GTTAAAGGCTTTGAACGCCGAAGCATCTATACTAAGCCAGTCATTT
HR550	CYP73A92opti_R213A_R	AAATGACTGGCTTAGTATAGATGCTTCGGCGTTCAAAGCCTTTAAC
***PpCYP73A* disruption construct (Gibson cloning)**		
HR487	pGEM-T_Easy_F	TCTATAGTGTCACCTAAATAGCTTG
HR488	pGEM-T_Easy_R	GCCCTATAGTGAGTCGTATTAC
HR489	CYP73A48_fragment5'_F	aatacgactcactatagggcggatccCTCTCATGTTTTGGTTGAATG
HR490	CYP73A48_fragment5'_R	gtcatagctgTTTCGCTCATCGACAAAATG
HR491	NPTII_CYP73A48_F	atgagcgaaaCAGCTATGACCATGATTACGC
HR492	NPTII_ CYP73A48_R	ggaatgtgcaTTGGGTAACGCCAGGGTT
HR493	CYP73A48_fragment3'_F	cgttacccaaTGCACATTCCATGCCAAC

HR494	CYP73A48_fragment3'_R	tatttaggtgacactatagaggatccTGTAATGTTGGTGCATTAAACAAATAA
HR501	CYP73A49_fragment5'_F	aatacgactcactatagggcggtaccTCGTCAAAGCTGAAATGCTTC
HR502	CYP73A49_ fragment5'_R	cggcaagcttGCCTGGGTCCCAAGGTTG
HR503	HPT_CYP73A49_F	ggacccaggcAAGCTTGCCGCCAAGGAT
HR504	HPT_CYP73A49_R	ttggcaacgaGGATCCCGATCTAGTAACATAGATG
HR505	CYP73A49_fragment3'_F	atcgggatccTCGTTGCCAACACTTTTTC
HR506	CYP73A49_fragment3'_R	tatttaggtgacactatagaggtaccAAAAATCCCATAACTTTGATACTTATTC
PpCYP73A:uidA **(Gibson cloning)**		
HR487	pGEM-T_Easy_F	TCTATAGTGTCACCTAAATAGCTTG
HR488	pGEM-T_Easy_R	GCCCTATAGTGAGTCGTATTAC
HR597	CYP73A48_GUS_fragment5'_F	aatacgactcactatagggcggatccTCTTGATGAGGAATGTAGGAGG
HR598	CYP73A48_GUS _ fragment5'_R	ttaagcctgcGACGGCAAGAGGTCTGGC
HR599	GUS_CYP73A48_F	tcttgccgtcGCAGGCTTAATGTTACGTC
HR600	GUS_CYP73A48_R	aaatgcaaacTCATTGTTTGCCTCCCTGCTG
HR601	CYP73A48_GUS _fragment3'_F	caaacaatgaGTTTGCATTTTTTGTGACAG
HR602	CYP73A48_GUS _fragment3'_R	tatttaggtgacactatagaggatccCAATGTTGGTTAATTTTCCTTC
HR591	CYP73A49_GUS_fragment5'_F	aatacgactcactatagggcgaattcACTTGAAGGTGTGTCAGGAG
HR592	CYP73A49_GUS _ fragment5'_R	ttaagcctgcAGCGATGGGTTTGCAGAC
HR593	GUS_CYP73A49_F	acccatcgctGCAGGCTTAATGTTACGTC
HR594	GUS_CYP73A49_R	gcgcttgagcTCATTGTTTGCCTCCCTGCTG
HR595	CYP73A49_GUS _fragment3'_F	caaacaatgaGCTCAAGCGCAATGTGGA
5HR96	CYP73A49_GUS _fragment3'_R	tatttaggtgacactatagagaattcTATTACAAAAACATAGCTAAAAGTACAACTATAC
**Double-strand DNA fragments (gBlocks)**		
HR1318	PcCYP73Aoptimized_CDS_attB12	ggggacaagtttgtacaaaaaagcaggcttcATGGCATGGGCCTGGCGTGCTTTAGCTGTCCTGTCAAGTGCGGCTATAGGCGCGGCCGCCGAAGAAGTAGCCGCTGCAGAGGCCGTACAGGCTTCCAGCAGTTCATTGGAGACCGCTCTATTGTCCCTTTTTGCCGTAGTCGTGGGTGGTATTCTTATCTTCCAATTGACCAGGAAAAAGCTGAACCTGCCACCTGGACCTGCTCCTGTGCCGATCTTCGGTAATTGGCTTCAGGTGGGCGATGATCTAAATCACAATAACTTAAGTGAACTAGCGAAAAAACACGGCGACTGCTTCTTATTGAGAATGGGTCAAAGAAACCTAACTGTCATTTCTAGCCCGGAGCTGGCTAAAGAAGTGCTTCATACACAAGGCGTTGAGTTTGGCAGTAGAACTAGGAATGTAGTACTTGATATTTTTACAGGTAATGGACAAGATATGGTTTTCACCGTCTATTCTGACCACTGGCGTAGAATGAGAAGGATAATGACAGTTCCATTTTTTACAAGCAAAGTAGTTACGTCCCTAAGATCTCGTTGGGAAAAGGAGGTTGATATTACTCTTGAGGACCTGAAAACGATGGAAGGGGCAAGTACTACTGGGATCGTGATCAGGAAGCGTTTGCAATTAATGATGTATAATTTTATGTACCTTATGATGTTTGACAGGCGTTTTCCGGGGGAAGACGACCCACTATACAACAAATTAAAGGTTCTTAACGCTGAGAGATCCCGTTTATCTCAGTCTTTTGAATTCAATTATGGGGATTTTATCCCCATACTGCGTCCTTTCTTGAGGAAGTATCTGGACCACTGCAGAGACATTAAACAGAAGCGTCTGGGCTTGTTCAAAGAACACTTCCTGACGGAAAGAAAGAAATTGCTAGATACCAAAGGACTAGGCGCGGAGAAACTGGGCATGGACCATGTGTTCGATGCTGAGAAGAACGGGGAGATAAACGAAGACAATGTGCTTTACATTGTGGAGAACATAAATGTCGCAGCAATCGAGACCACCCTATGGTCTATCGAATGGGGGATCGCCGAACTTGTCAACCACCCGAAAATCCAGGCGAAGTTGAGGGAGGAGTTAGATACAGTACTTGGCAAAGGAGTTCCTGTTACCGAACCGGATGTTCAGAGTGGCAAGTTACCTTACCTAAGTGCAGTCGTCAAGGAAACATTGAGGTTGCACATGGCAATTCCTTTGTTGGTACCACATATGAATCTTACTCAAGCGAAGTTAGGGGACTACGACATTCCGGCTGAATCTCGTATCTTGGTGAACGCTTGGTGGTTAGCGAATAATCCCGACCTTTGGAAAAAACCTGAGGAATTCCGTCCAGAAAGATTCTTAGAGGAGGAAAAAGGGGTGGAAGCGAACGGCAACGATTTTCGTTTTCTGCCCTTCGGTGTCGGTAGGCGTAGTTGCCCGGGCATCATCTTGGCGCTTCCTATCTTGGGCTTAGTCCTAGGCAAACTGGTCCAGACATTTGAACTAAGCACGCCCCCAGGTGTCGATAAGGTGGACACAGAAGGACAGGCGGGGCAGTTCAGTCTTCGTATAAAAACTCATTCCACTGTTGTTGTGAAACCGCGTGCTTGAgacccagctttcttgtacaaagtggtcccc
HR1098	kfl00038_0230 _CDS_attB12	ggggacaagtttgtacaaaaaagcaggcttcATGGACACTTTTACTGAGGGCTTGAAAACCAATCCAGTCAATGCATTTCTGATCTCCTTGGTGGTTGTCTTTCTGACCCCAATCTTGTGGACTATCGTGCACCAATATGTTAAGGTGCTGAGAGCCCGGGGCATTCCCCCCGGCCCTGCTCCACTGCCAGTCTTGGGCAACCTGGGGGAAATTCTTTCGAGCCCCAAGCGCTTTGATGAGTGGTGCCTTCAAAAGGCCAAGGCATATGGCGGCATGTTCAGCTTCTGGATGGGAGATCAGCTGTTTGTGTACGTCAGCTCGTATGAAGCGGTCAAGGAGGTCCTGGTTACGAAGGATAAAGAGTTTGCATCGAGGCCCCTGATGCCTGAGCGTGTCGCCCTCACTTTGGGGCCGAAGAGCATCTTCATGTCAAGCTACGGGGAATACTGGCGCCAGGCTCGTAAGCTGTCGGTCCTCCATCTACTCTCCAACAAGGCTGTCAAGACCAACGCCTCAGTTCGTGATGAAGAACTTAACACCCTCATCGGCCATCTGAAGGAAGAGTGCAAGCGGGAGGGGGGCATTGTGGAGCCGCGCGAACACATCATCAGGACCAATCTCAACACCATGATGCTCCCACTGTTCGGCATCCGTTTTCCCGCCCCCACGGAGCCGGGCTTTAACGAGAGGCGCGAGGCGCTCTACGCGAGCATTCAGGAGAGCTTCCGCCAGATGGGCGACTTTGATTGGGGAACCGTGCTGCCCGCGCTTGCGCCGGTCGTGAACCGTGACGTCAGGAAGACGACAGCGTTTAGGGACAGGGAGCTGGAGCTTCTGATTGAGGAGCACCGCGCCGCCAAGGAGAAGGCGGGGGCCAGCTTCGTGGCGCGGGATATGGTTGACGTACTGCTGACGATACCCGAAACGGAGGACCTGACGCTCAAGAACCTGATCTACATGCTTCTCGATCTGCTGAACGCGGCGGTGGACACGAGCGGCGTCTCCATCGAGTGGACGCTAGCCGAGCTGACCCGACGGCCGGAAATCCGGAAACGGTTGCAAGCGGAGCTGGACAGTGTAATTGGTCGGGAGAGACCGGTGCAAGAAGGGGACATCGAGAATTTGCCATATCTCCGAGCAGTCATCAAGGAGCATTTTCGATACCGGGGCGTCGGTCCCTTCCCCGGACCGCATCTGAATGAAGTCGAGACGACGGTCCAGGGGTATCGGATTCCCGCCAAAACGCAGGTGATGGTGCACACGCGGGGCTTGTCGCGGGATCCCTCTGTCTTCTCCCGGGCGGATGAGTTTCTCCCCGACCGCTTTCTCGCAGAGGACGTCGACATCACTGGAAAGGACCTCCGGGTGCTACCTTTTGGCGCAGGTCGGAGGGGGTGTCCTGGCATCGTTCAAGGCCTAGCCGTCGTACATCTGGCGGTGGCCCGGATCGTTCAAGCCTTCGACTTTGAGCTGATCGACGGAAAAGACATCGACATGGAGGAGCGAAACTGGGGCCTGCAAGTCTCATTGCGCACGCCGCTGAAAGCCCGGTTTACGCCCCGTTAAgacccagctttcttgtacaaagtggtcccc

Appendix Table S4. List of multiple reaction monitoring (MRM) methods used for targeted analysis by UHPLC-MS/MS.

Molecule	Ionization mode	Precursor ion	Collision energy	Product ion
cinnamic acid	positive	149.0	21.0	103.1
p-coumaric acid	negative	163.0	9.0	119.1
caffeic acid	negative	179.0	18.0	135.1
ferulic acid	positive	195.0	10.0	177.0
p-coumaroyl-threonate	positive	283.0	10.0	147.0
p-coumaroyl-shikimate	positive	320.9	8.0	147.0
caffeoyl-threonate	positive	299.0	38.0	89.0
caffeoyl-shikimate	positive	337.4	13.0	163.0
rosmarinic acid	negative	359.1	8.0	161.0

Appendix Table S5. List of quantification masses and molar response factors used for quantitative analysis of cuticular monomers by GC-TOFMS. IS, internal standard; TMS, Trimethylsilyl group.

**Analyte**	**Quantification mass (±500ppm)**	**Molar response factor (relative to IS)**
ribitol, 5TMS (IS for glycerol)	73.08	1.00
glycerol, 3TMS	73.08	1.05
C17:0, 1TMS (IS for FAMEs)	74.06	1.00
t-cinnamate, methyl ester	131.06	2.11
p-coumarate, 1TMS, methyl ester	73.07	2.78
caffeate, 2TMS, methyl ester	73.07	0.84
ferulate, 1TMS, methyl ester	250.08	1.23
16-OH C16:0, 1TMS, methyl ester	75.05	2.13
10,16-diOH C16:0, 2TMS, methyl ester	73.07	1.52
1,9,18-triOH C18:0, 3TMS	73.07	*1.52
C14:0, methyl ester	74.06	0.58
C16:0, methyl ester	74.06	0.61
C18:0, methyl ester	74.06	0.61
C18:1, methyl ester	55.09	1.16
C18:2, methyl ester	67.08	3.74
C18:3, methyl ester	79.07	0.76
C20:0, methyl ester	74.06	0.62
C20:4, methyl ester	79.08	#0.62
C22:0, methyl ester	74.06	0.62
C24:0, methyl ester	74.06	0.64

*response factor of 10,16-diOH C16:0, 2TMS, methyl ester; #response factor of C20:0.
