# Supplementary figures and images for: An ancient role for CYP73 monooxygenases in phenylpropanoid biosynthesis and embryophyte development
Source: EMBO J. 2024 Aug 1;43(18):13. doi: 10.1038/s44318-024-00181-7 (PMC11405693; doi:10.1038/s44318-024-00181-7)

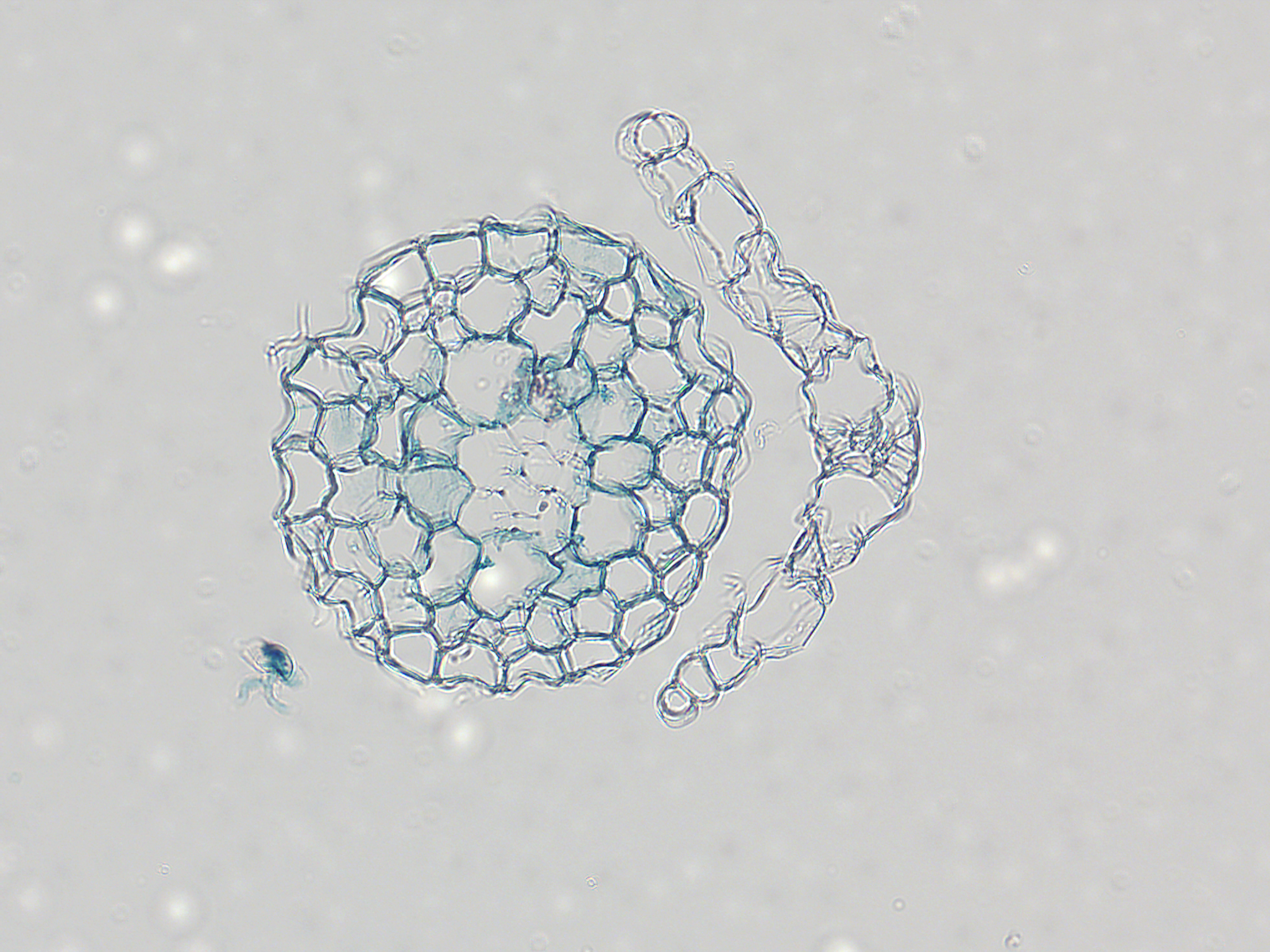

Supplement: Supplementary file 11 — Source data Fig. 3 [file 44318_2024_181_MOESM11_ESM.zip › Figure 3/3E/PpCYP73A48-uidA_cross-section.tif]

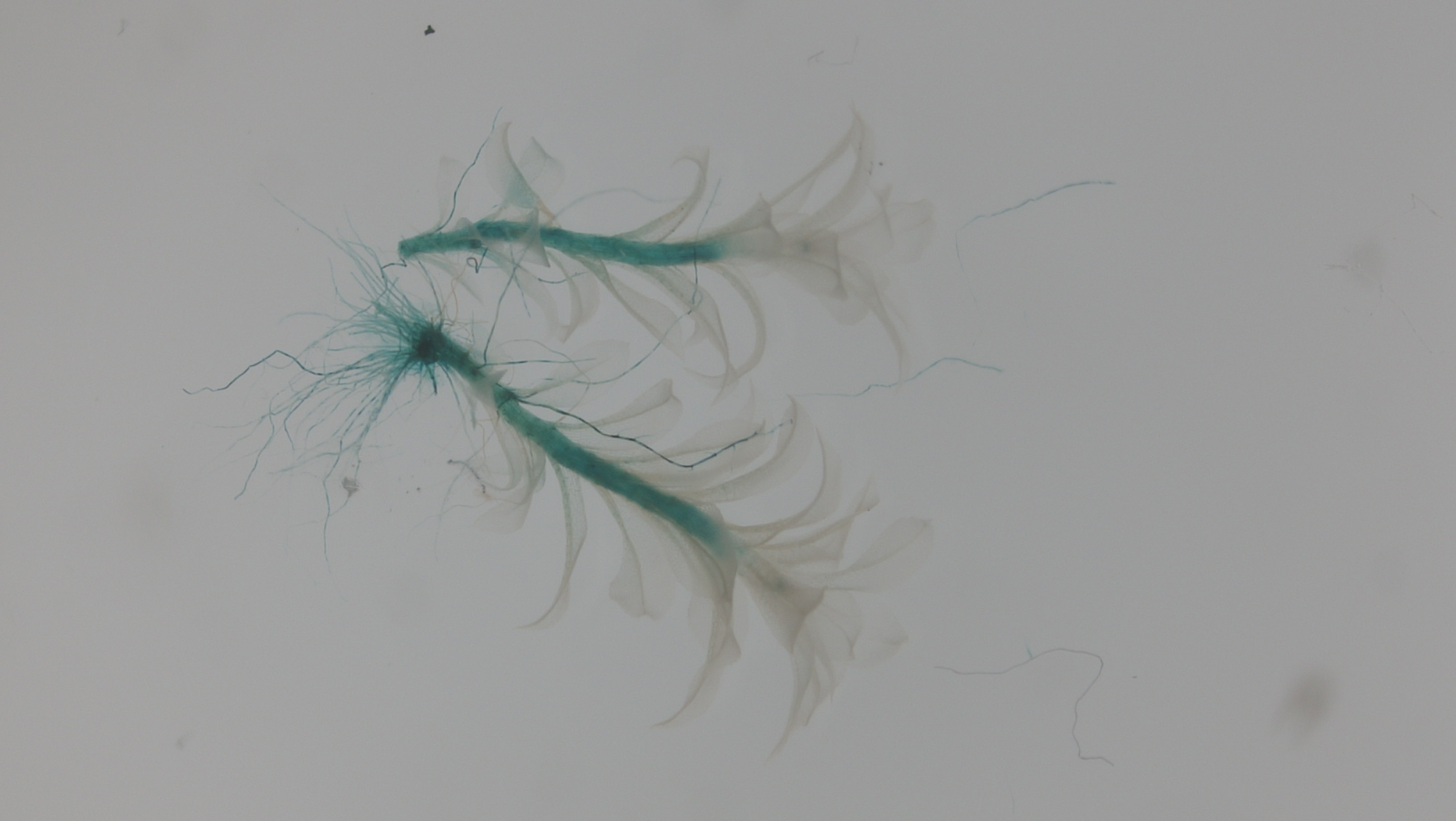

Supplement: Supplementary file 11 — Source data Fig. 3 [file 44318_2024_181_MOESM11_ESM.zip › Figure 3/3E/PpCYP73A48-uidA_whole_gametophore.JPG]

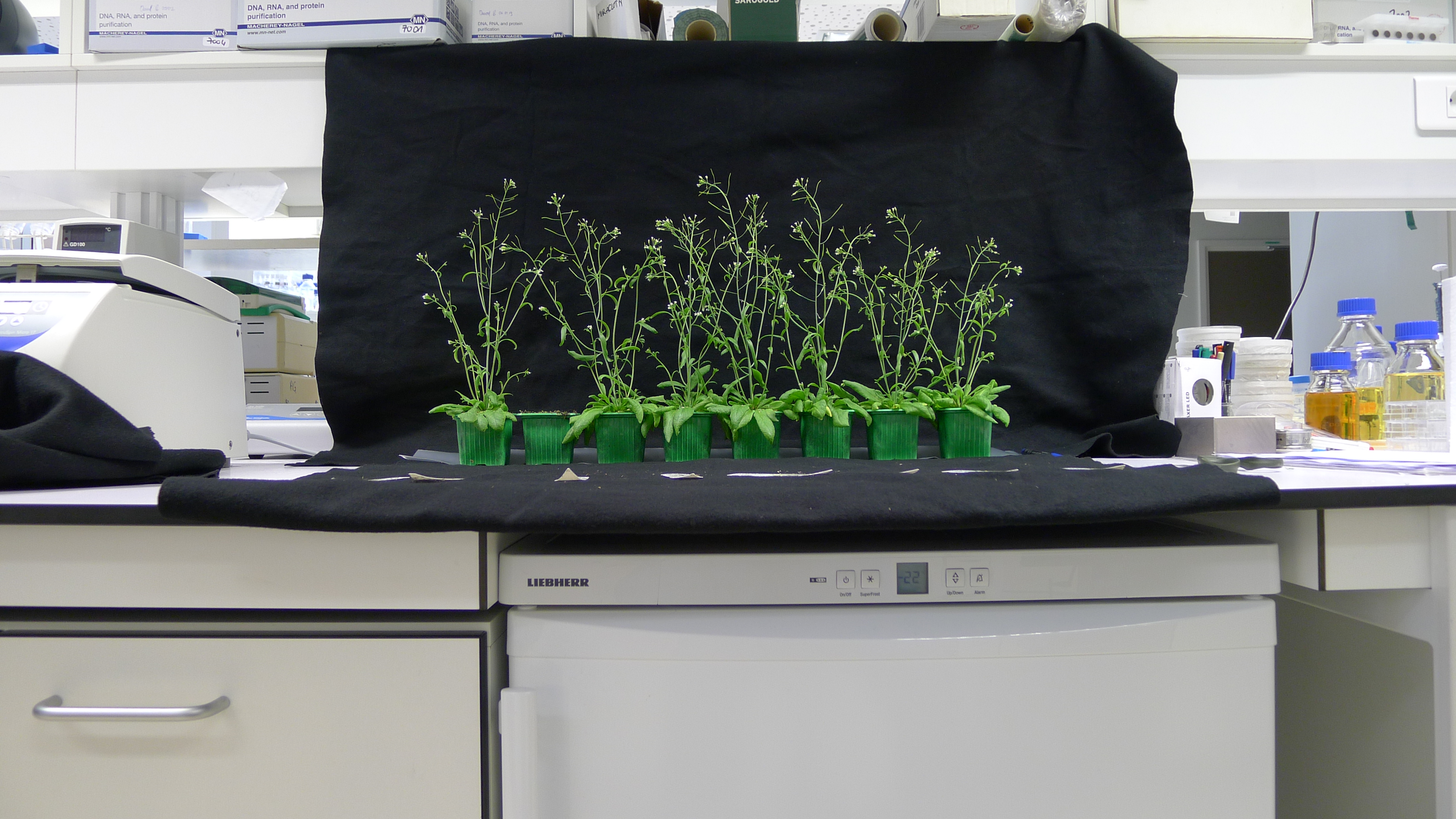

Supplement: Supplementary file 11 — Source data Fig. 3 [file 44318_2024_181_MOESM11_ESM.zip › Figure 3/3D/6-week-old_Arabidopsis_plants.JPG]

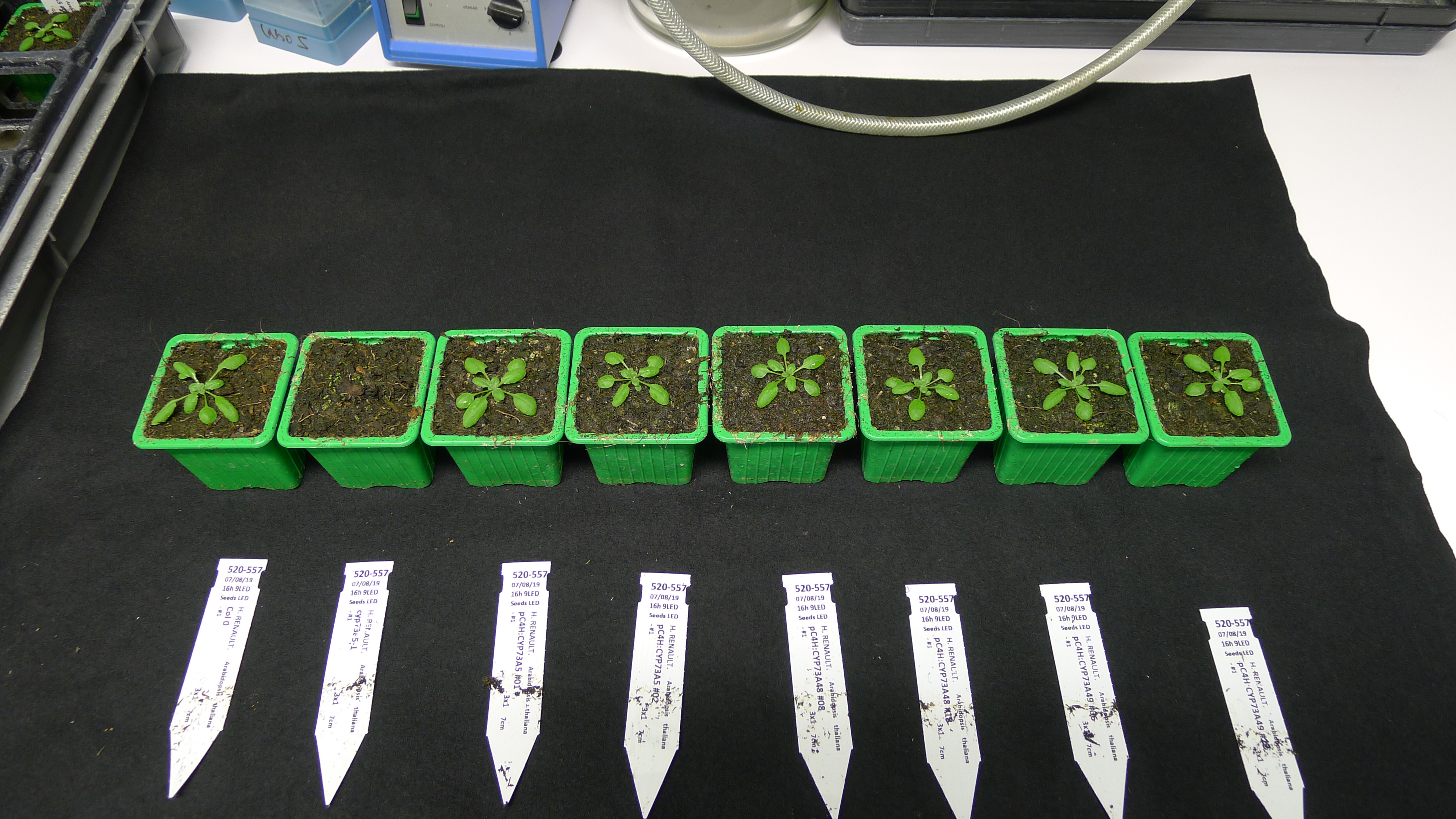

Supplement: Supplementary file 11 — Source data Fig. 3 [file 44318_2024_181_MOESM11_ESM.zip › Figure 3/3D/3-week-old_Arabidopsis_plants.JPG]

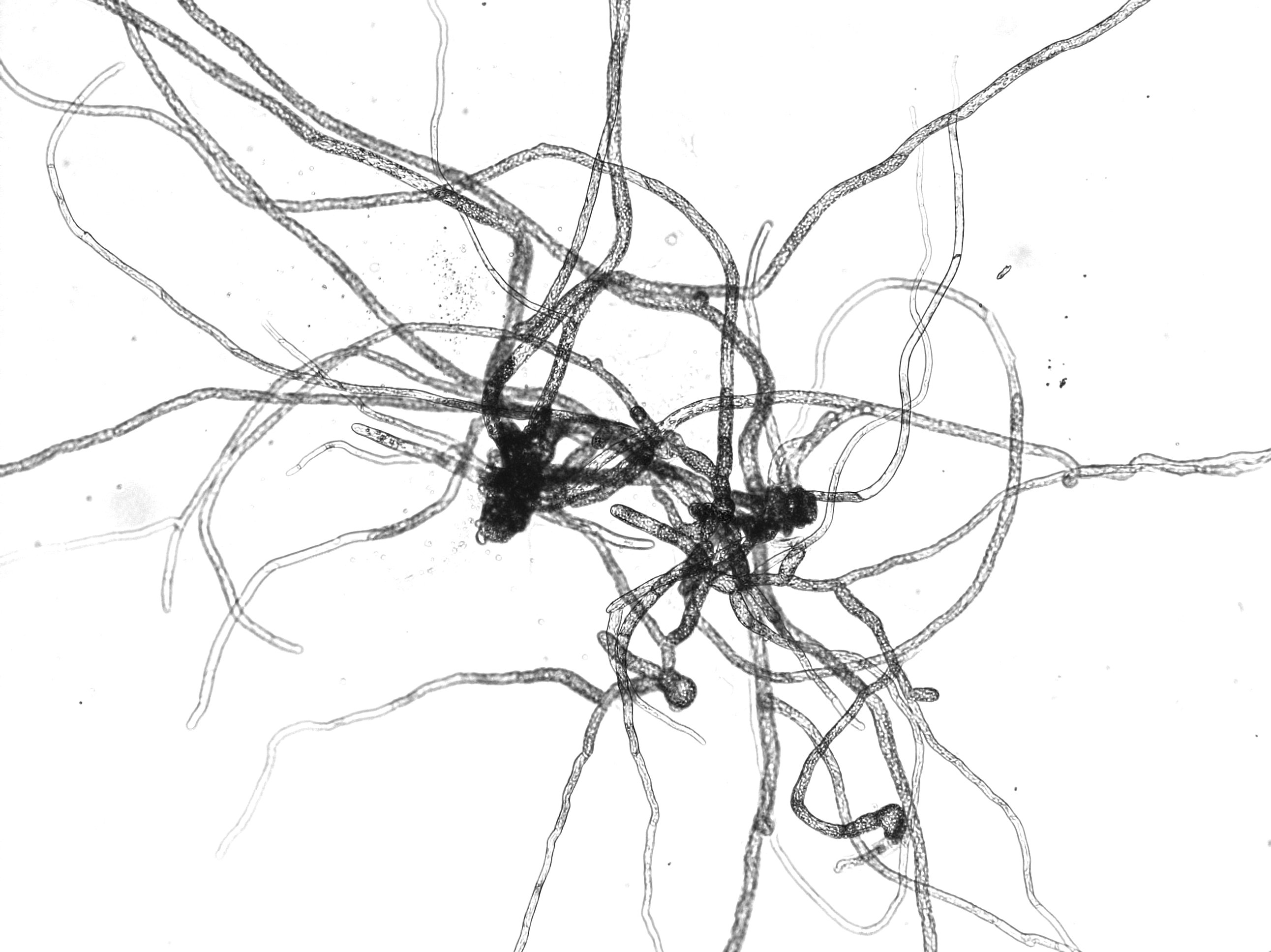

Supplement: Supplementary file 11 — Source data Fig. 3 [file 44318_2024_181_MOESM11_ESM.zip › Figure 3/3H/╬öCYP73A48-CYP73A49_mock.jpg]

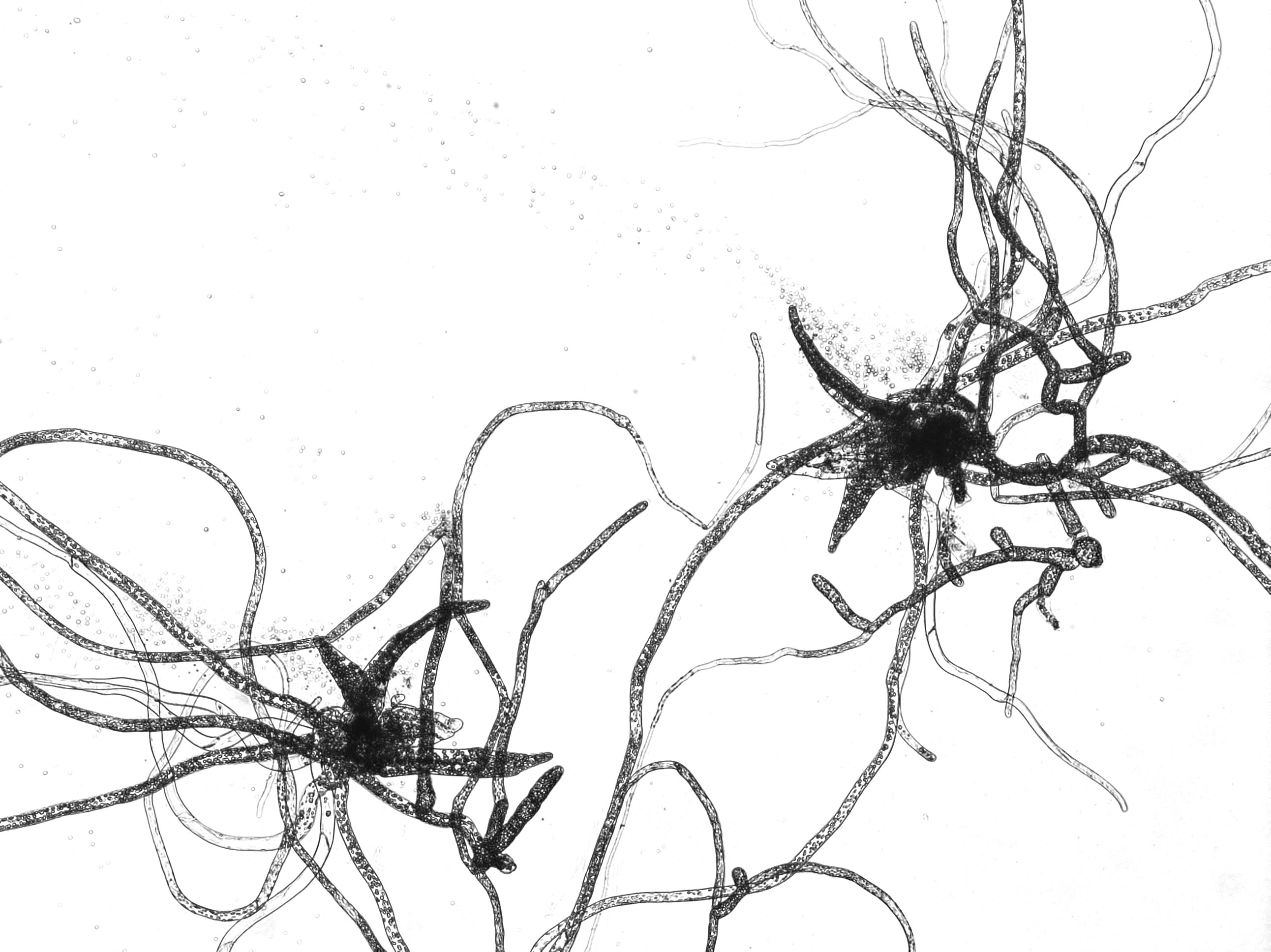

Supplement: Supplementary file 11 — Source data Fig. 3 [file 44318_2024_181_MOESM11_ESM.zip › Figure 3/3H/╬öCYP73A48-CYP73A49_pcoumaric_acid.jpg]

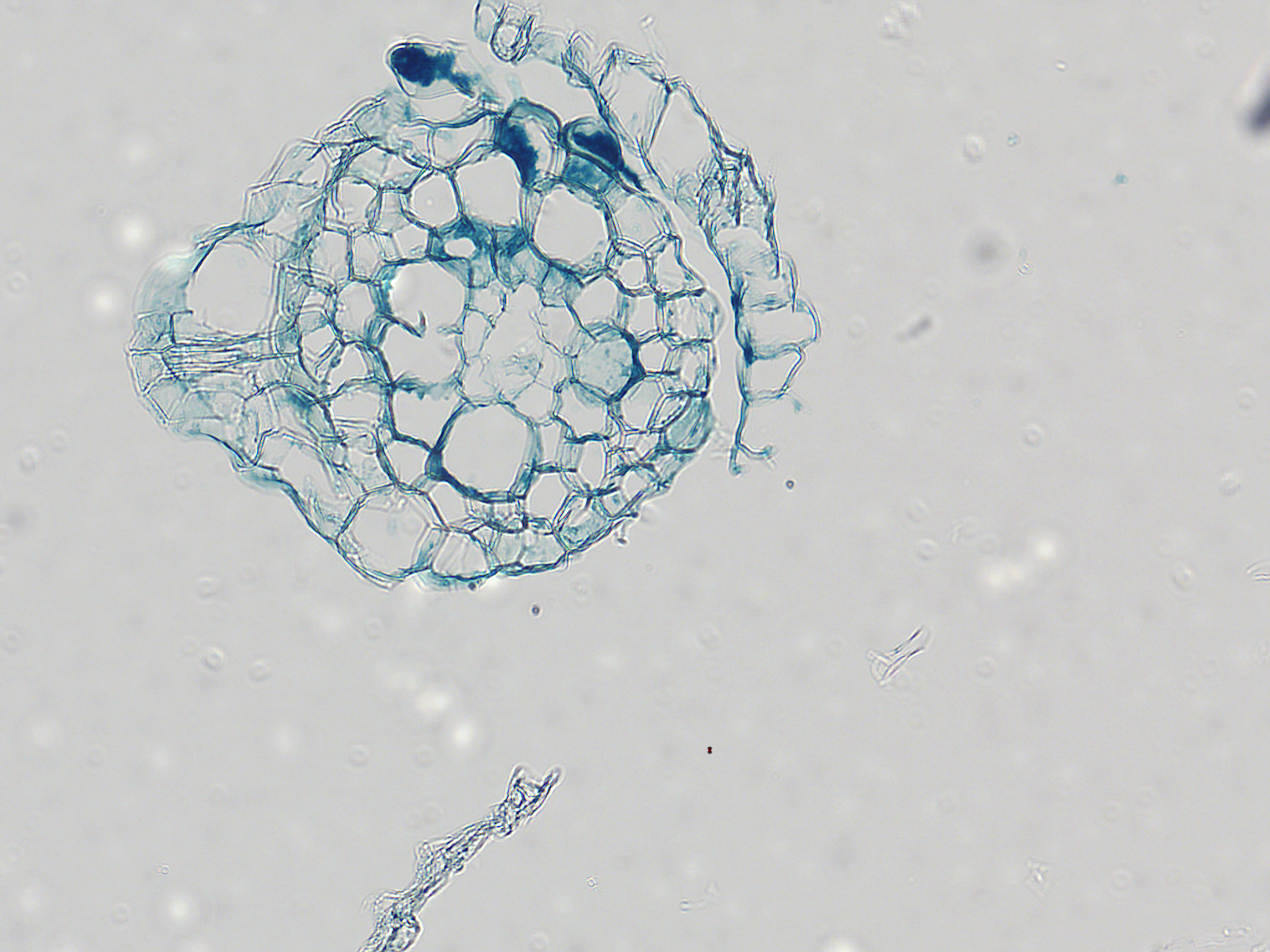

Supplement: Supplementary file 11 — Source data Fig. 3 [file 44318_2024_181_MOESM11_ESM.zip › Figure 3/3F/PpCYP73A49-uidA_cross-section.tif]

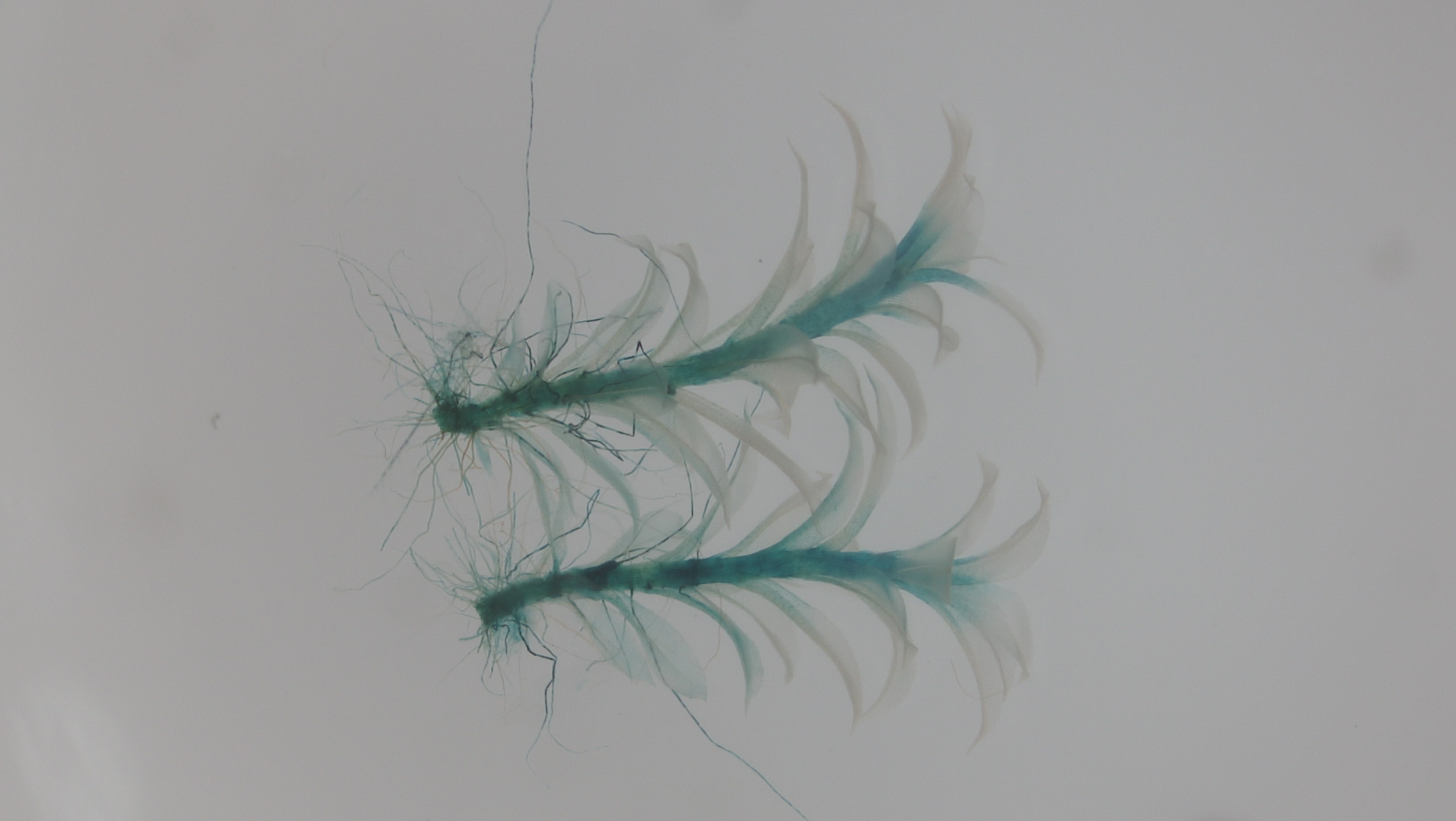

Supplement: Supplementary file 11 — Source data Fig. 3 [file 44318_2024_181_MOESM11_ESM.zip › Figure 3/3F/PpCYP73A49-uidA_whole_gametophore.JPG]

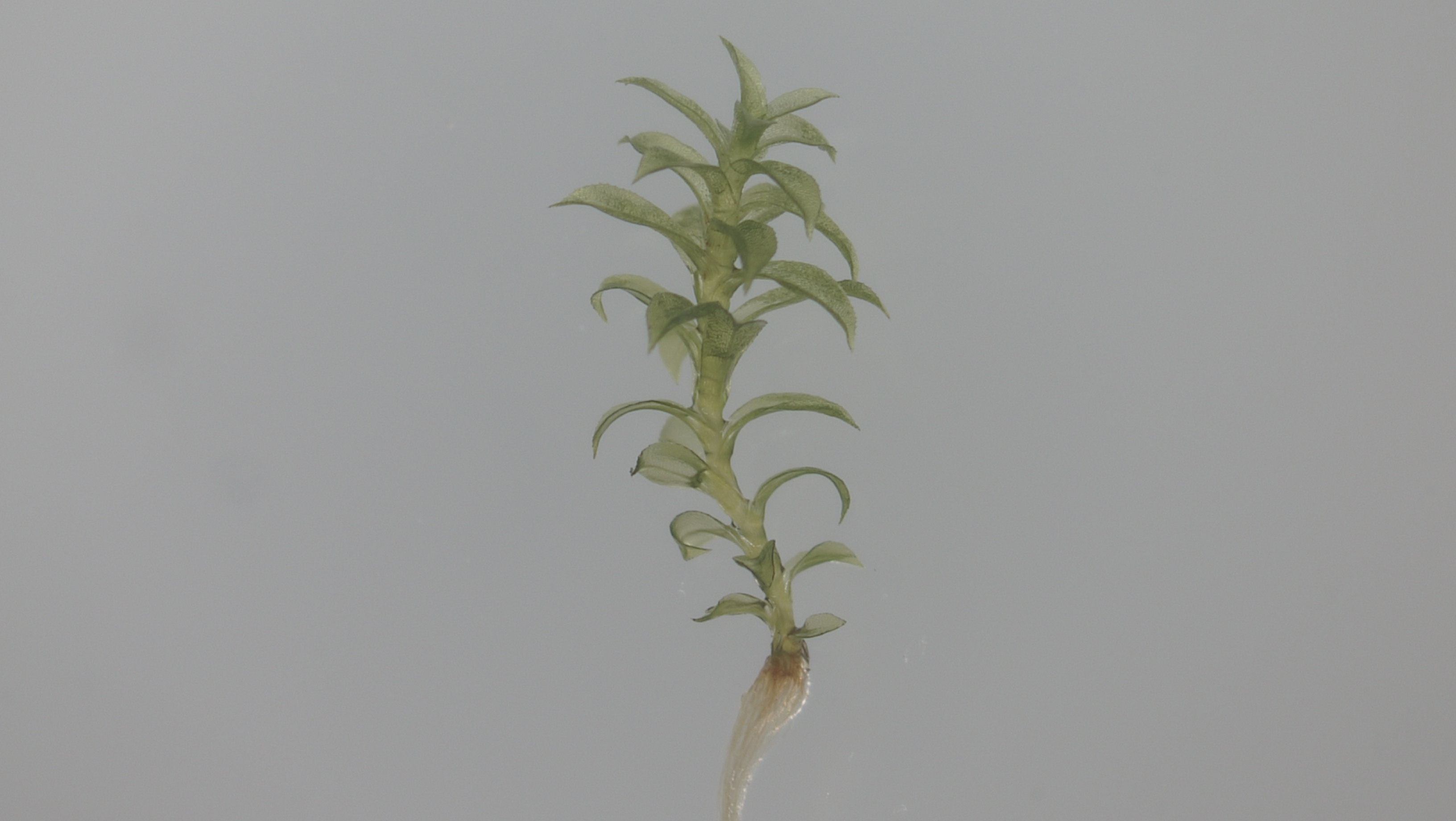

Supplement: Supplementary file 11 — Source data Fig. 3 [file 44318_2024_181_MOESM11_ESM.zip › Figure 3/3G/╬öCYP73A48_gametophore.JPG]

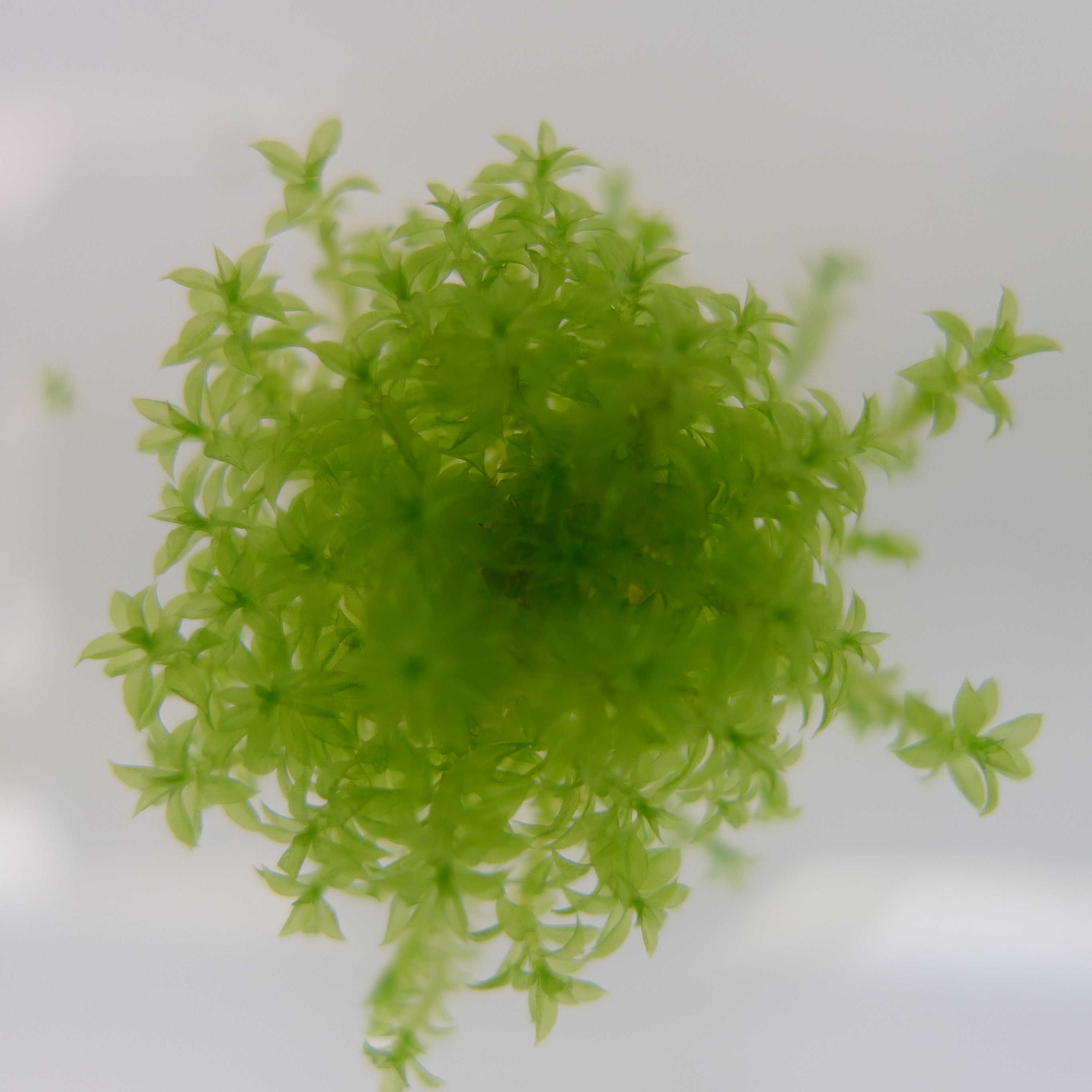

Supplement: Supplementary file 11 — Source data Fig. 3 [file 44318_2024_181_MOESM11_ESM.zip › Figure 3/3G/╬öCYP73A48_colony.JPG]

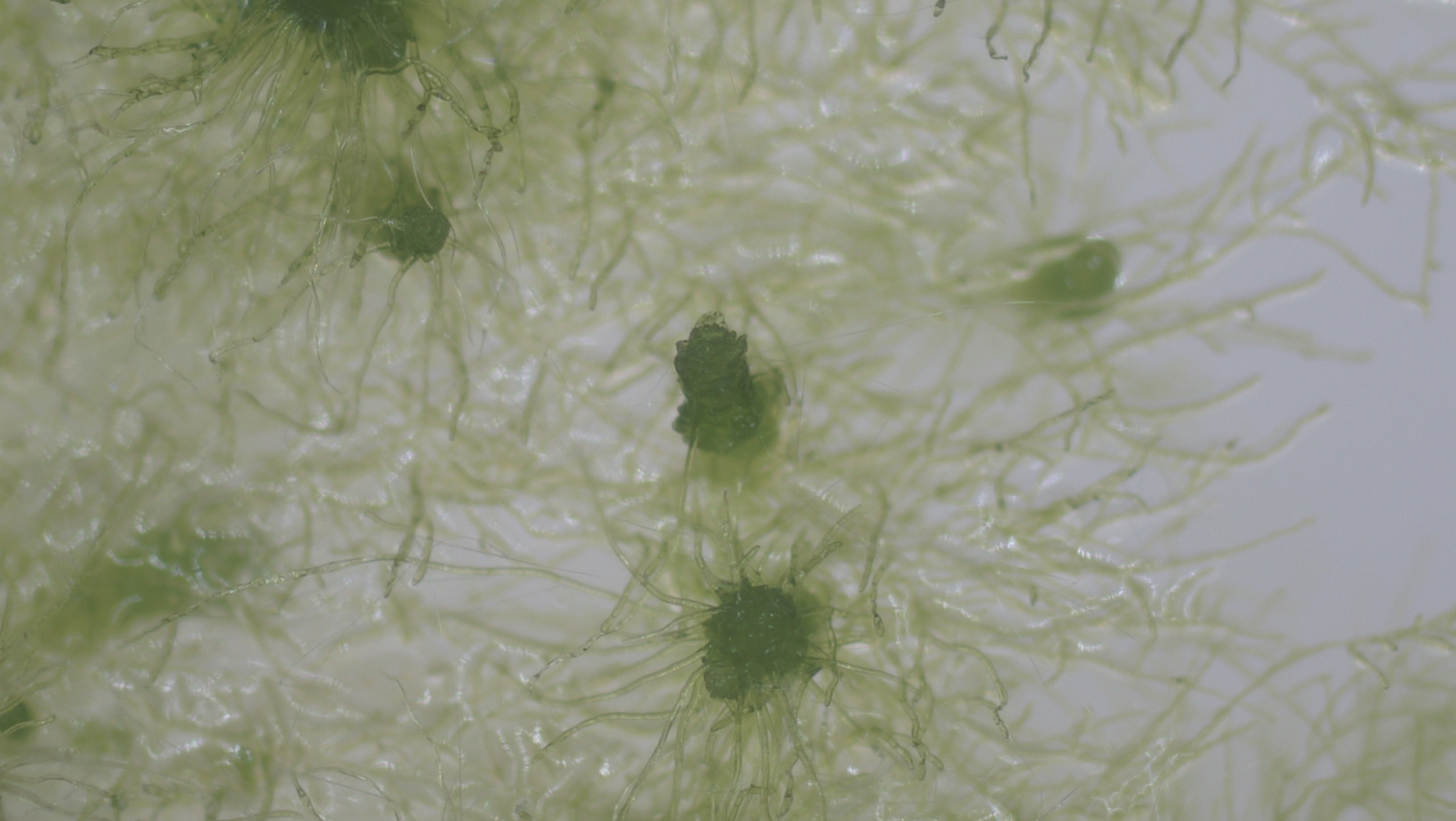

Supplement: Supplementary file 11 — Source data Fig. 3 [file 44318_2024_181_MOESM11_ESM.zip › Figure 3/3G/╬öCYP73A48-CYP73A49_gametophore.JPG]

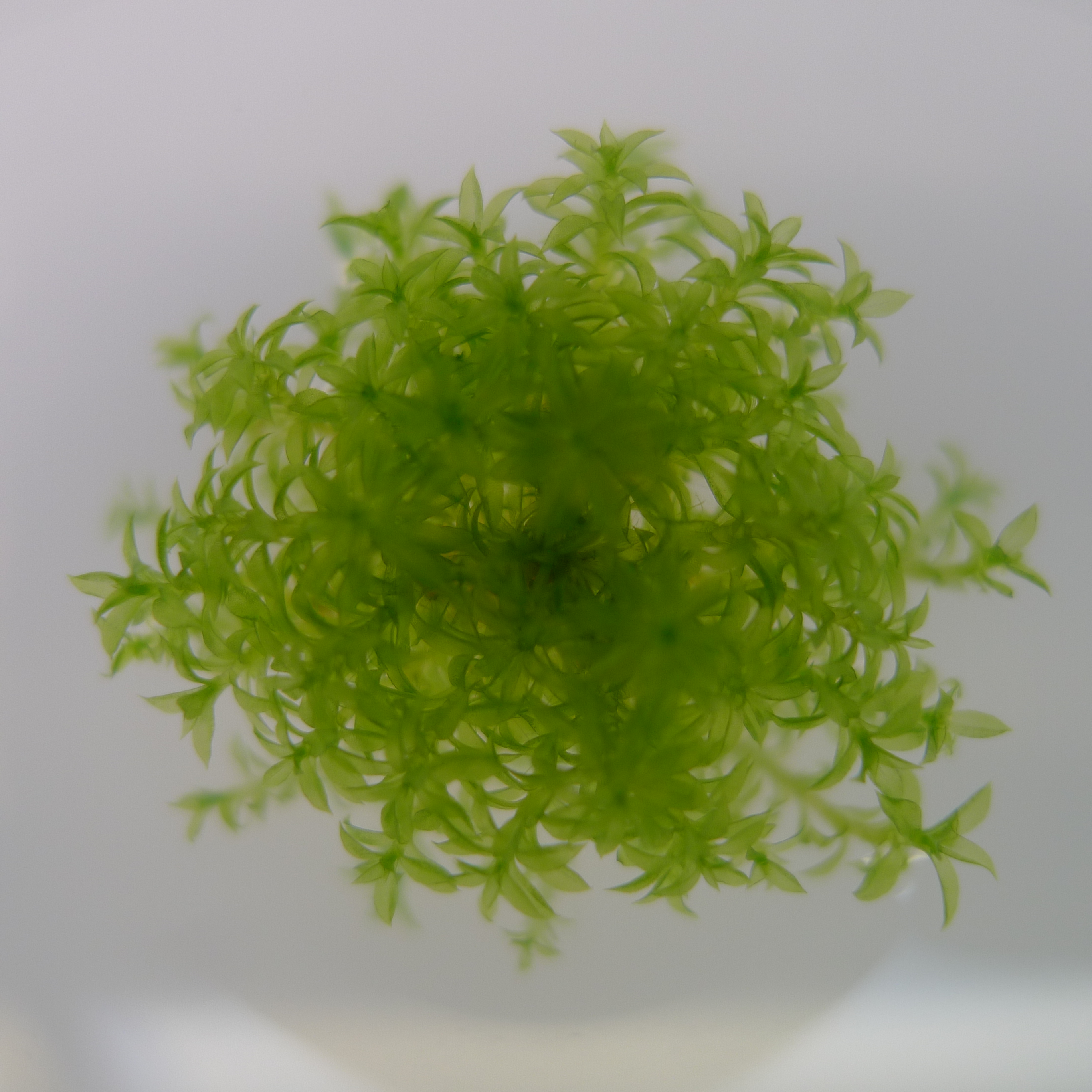

Supplement: Supplementary file 11 — Source data Fig. 3 [file 44318_2024_181_MOESM11_ESM.zip › Figure 3/3G/Wild_type_colony.JPG]

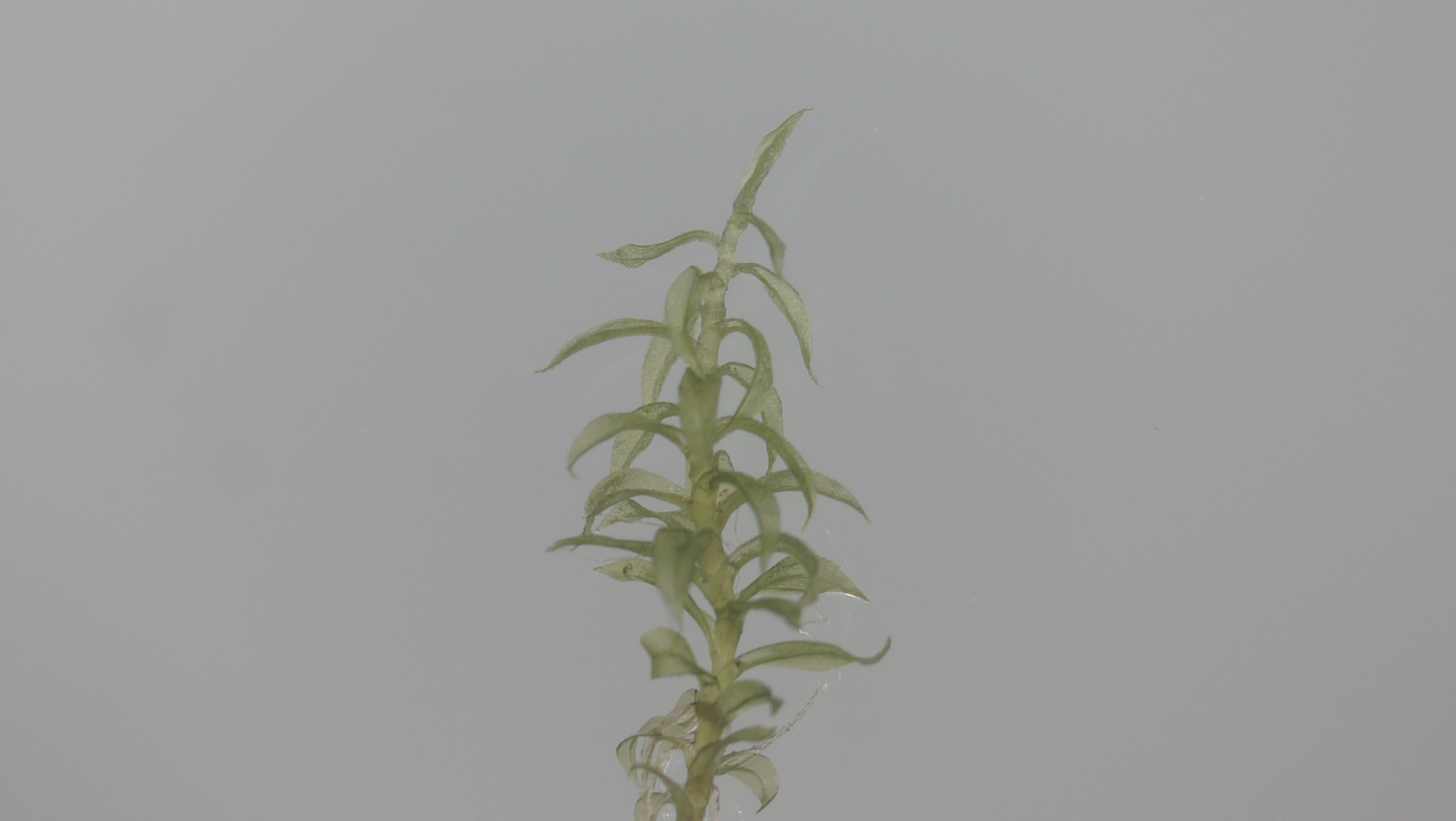

Supplement: Supplementary file 11 — Source data Fig. 3 [file 44318_2024_181_MOESM11_ESM.zip › Figure 3/3G/╬öCYP73A49_gametophore.JPG]

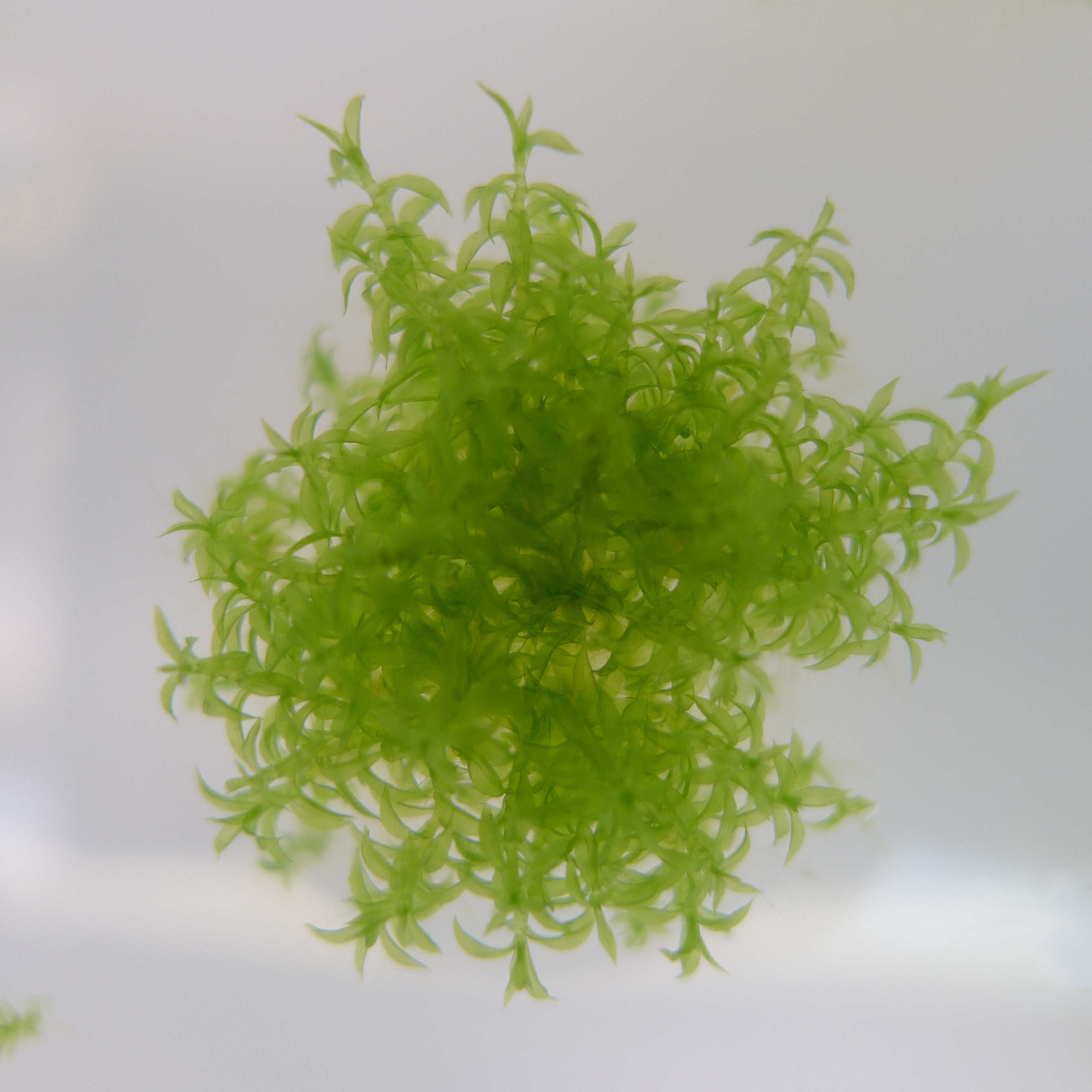

Supplement: Supplementary file 11 — Source data Fig. 3 [file 44318_2024_181_MOESM11_ESM.zip › Figure 3/3G/╬öCYP73A49_colony.JPG]

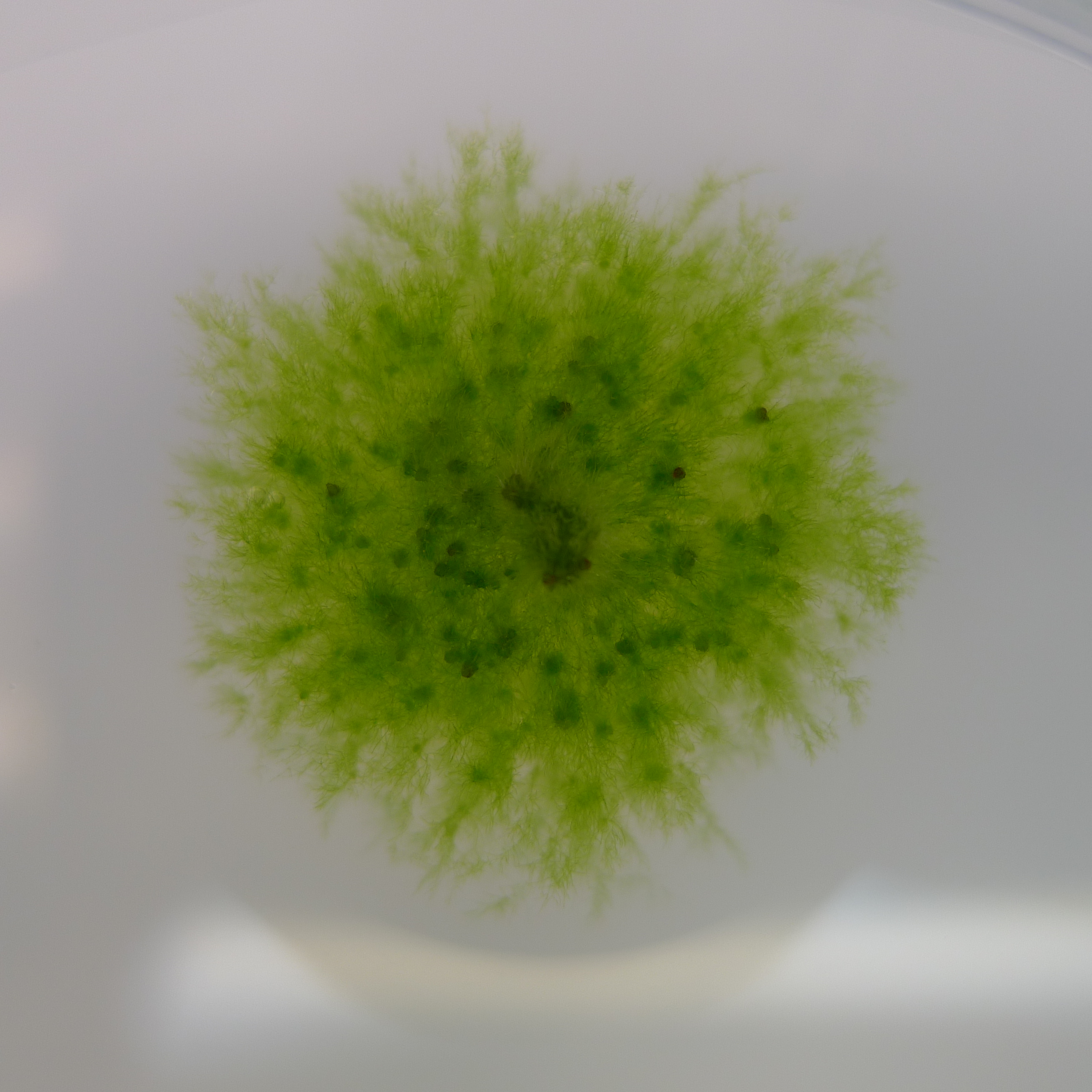

Supplement: Supplementary file 11 — Source data Fig. 3 [file 44318_2024_181_MOESM11_ESM.zip › Figure 3/3G/╬öCYP73A48-CYP73A49_colony.JPG]

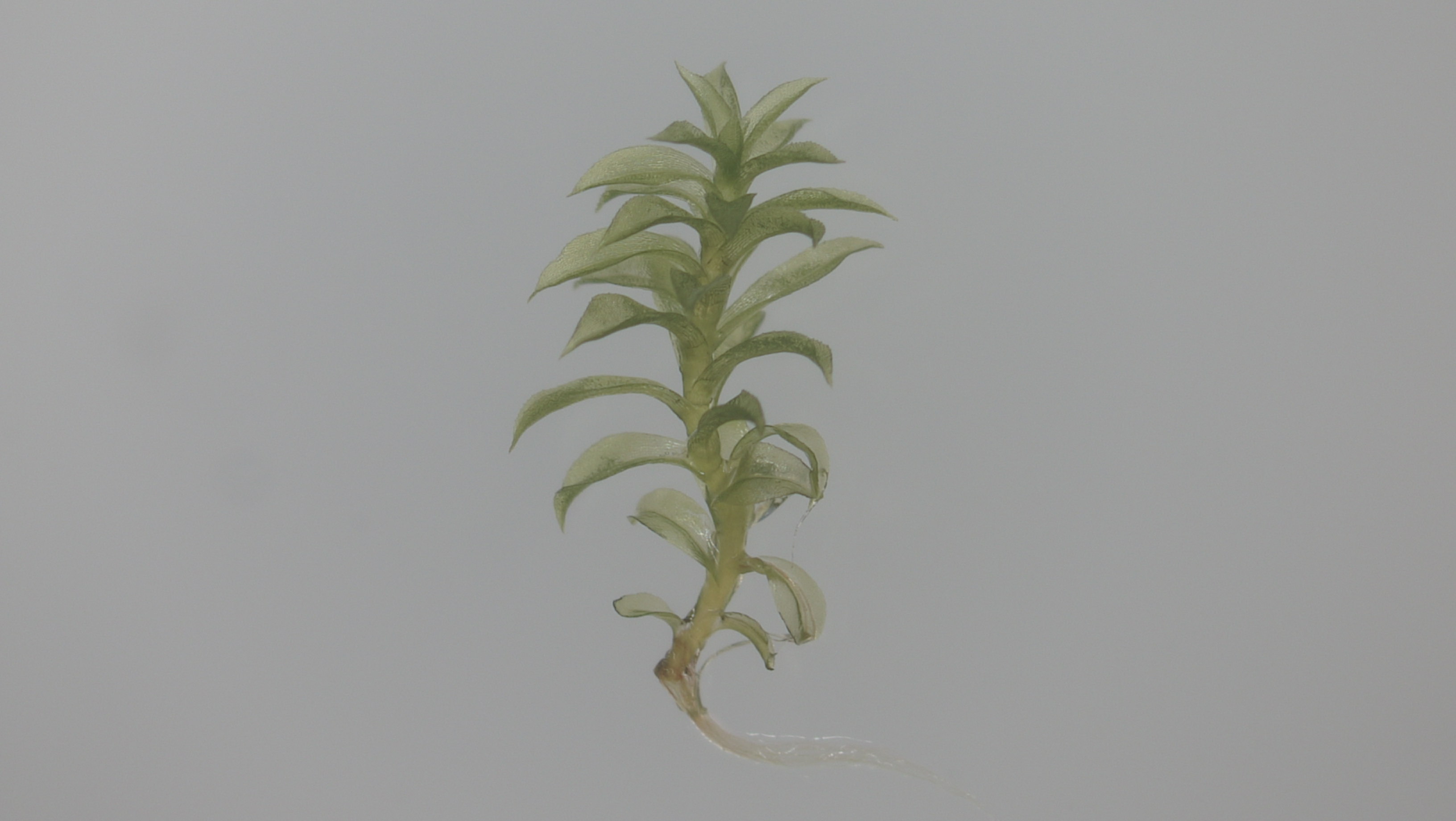

Supplement: Supplementary file 11 — Source data Fig. 3 [file 44318_2024_181_MOESM11_ESM.zip › Figure 3/3G/Wild-type_gameophore.JPG]

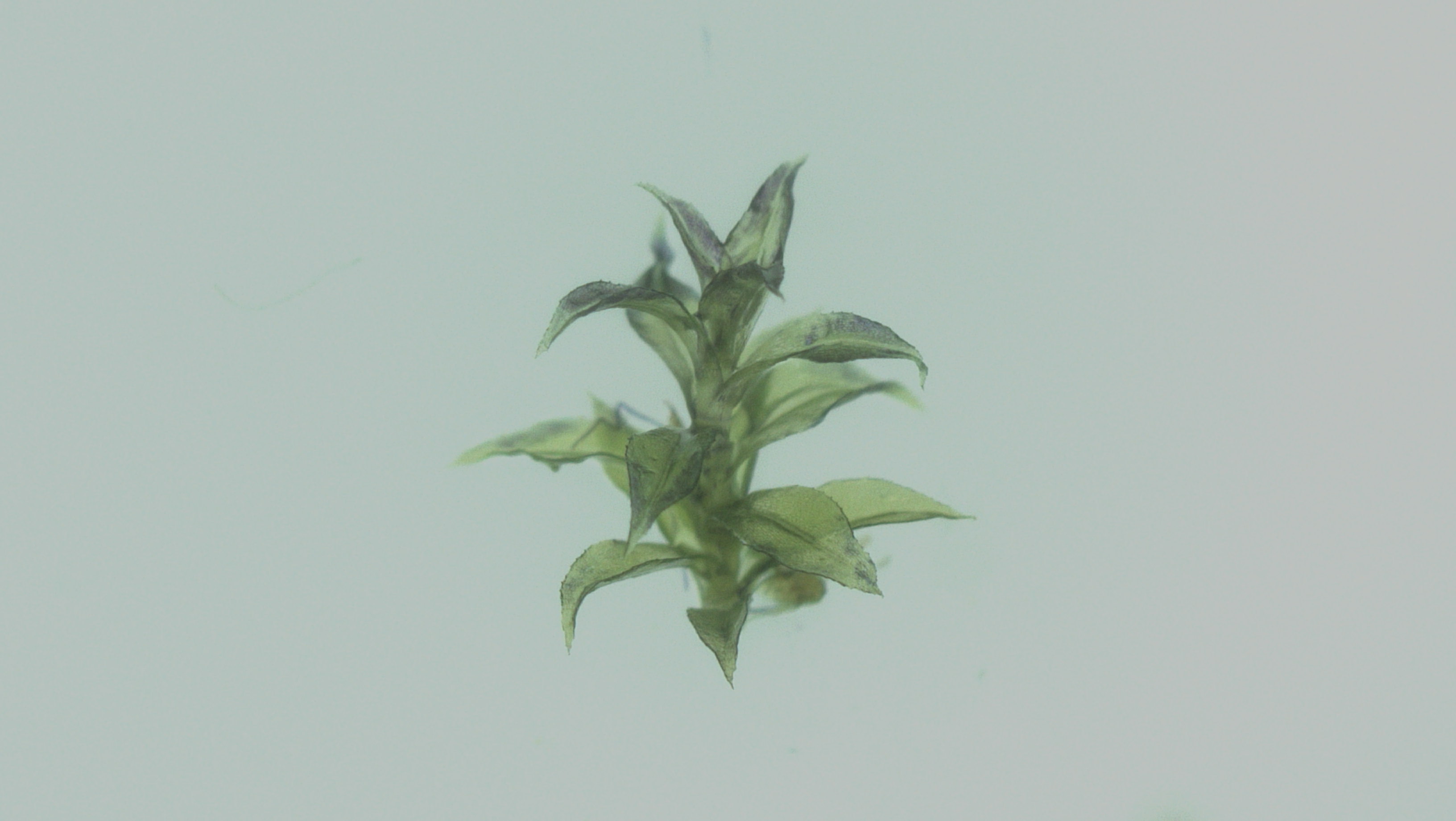

Supplement: Supplementary file 12 — Source data Fig. 4 [file 44318_2024_181_MOESM12_ESM.zip › Figure 4/4F/╬öPpCYP73A49_#03_Toluidine_blue.JPG]

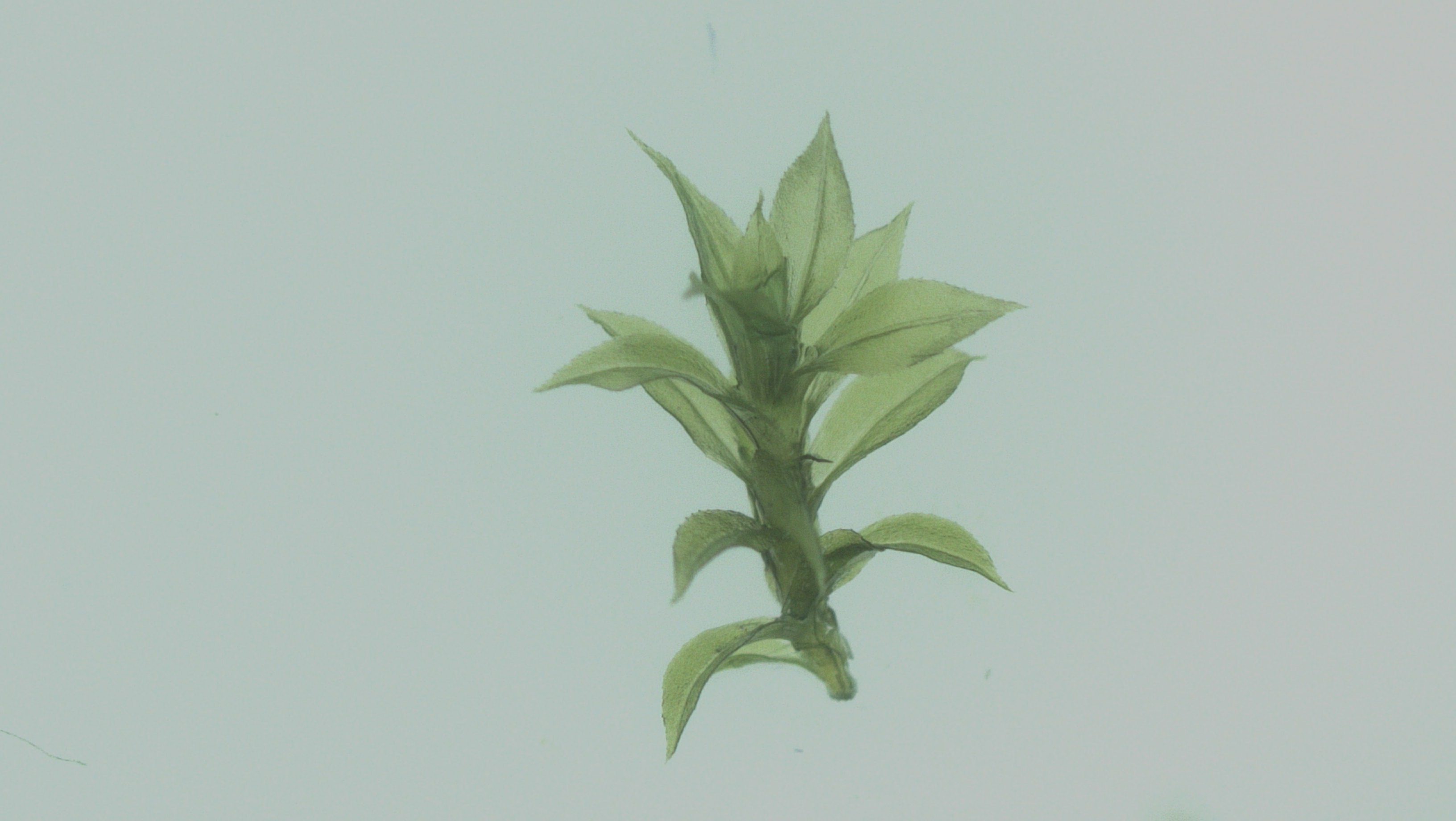

Supplement: Supplementary file 12 — Source data Fig. 4 [file 44318_2024_181_MOESM12_ESM.zip › Figure 4/4F/╬öPpCYP73A48_#23_Toluidine_blue.JPG]

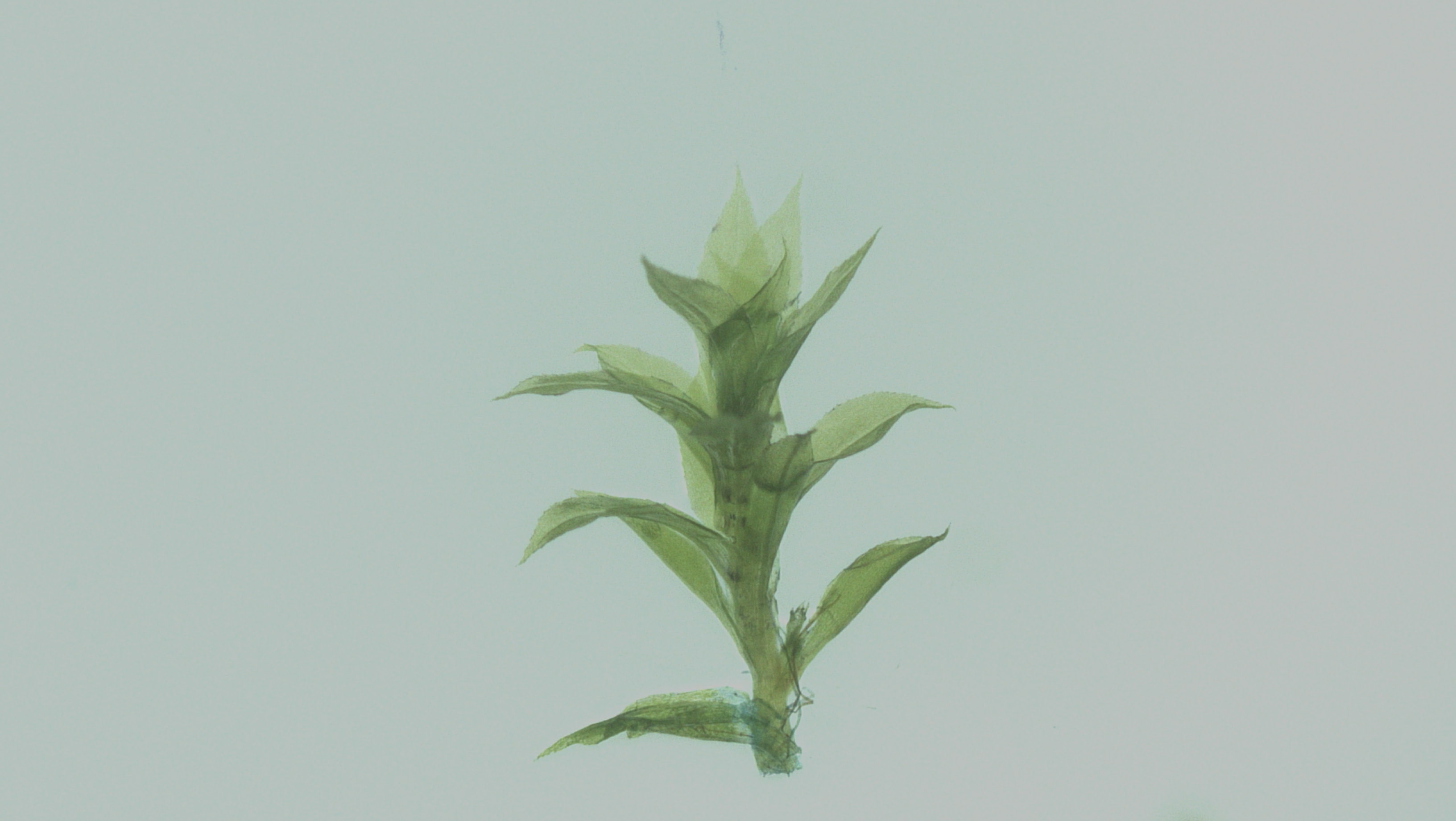

Supplement: Supplementary file 12 — Source data Fig. 4 [file 44318_2024_181_MOESM12_ESM.zip › Figure 4/4F/Wild_type_Toluidine_blue.JPG]

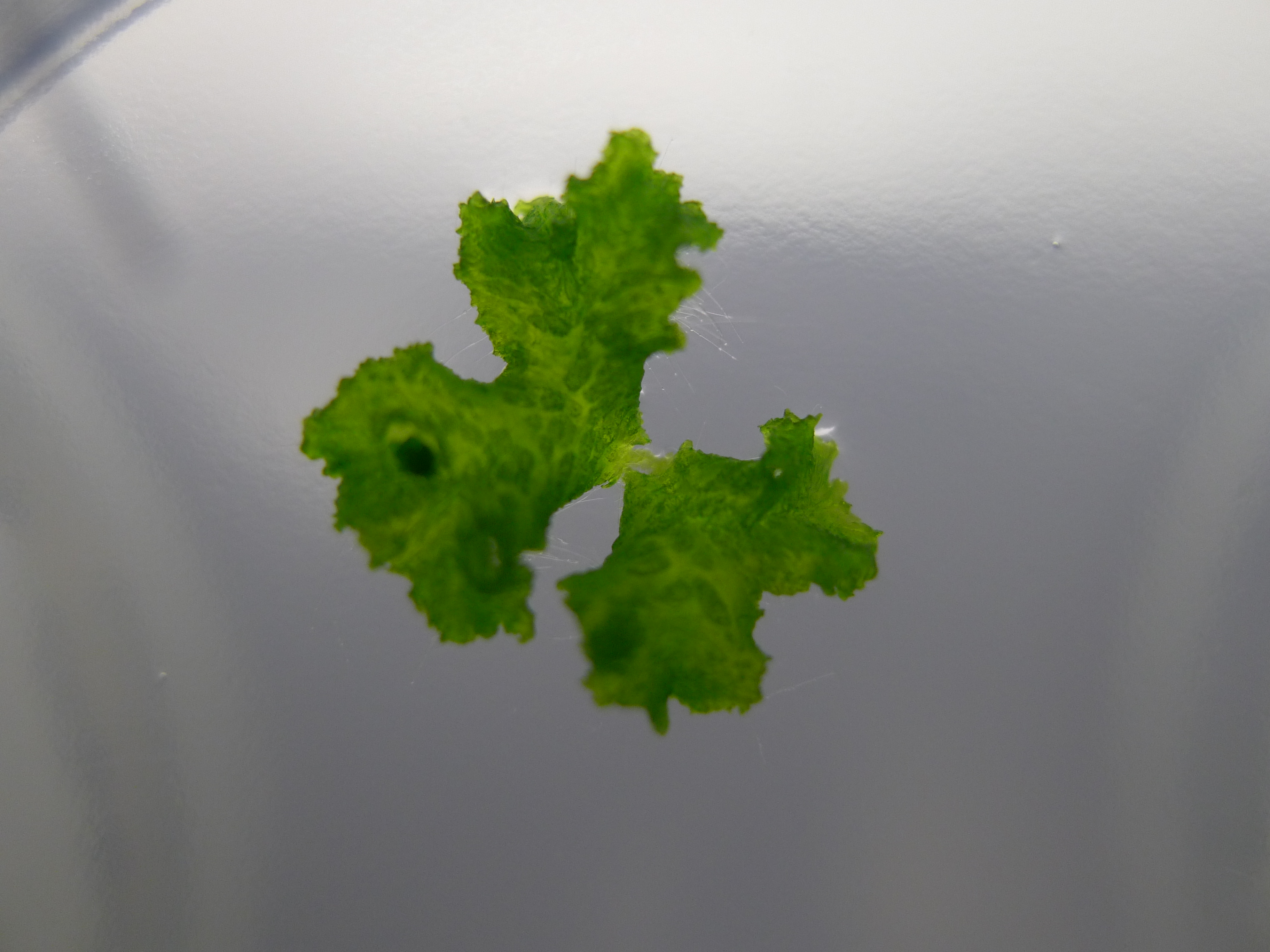

Supplement: Supplementary file 13 — Source data Fig. 5 [file 44318_2024_181_MOESM13_ESM.zip › Figure 5/5C/Mpcyp73a1-1.JPG]

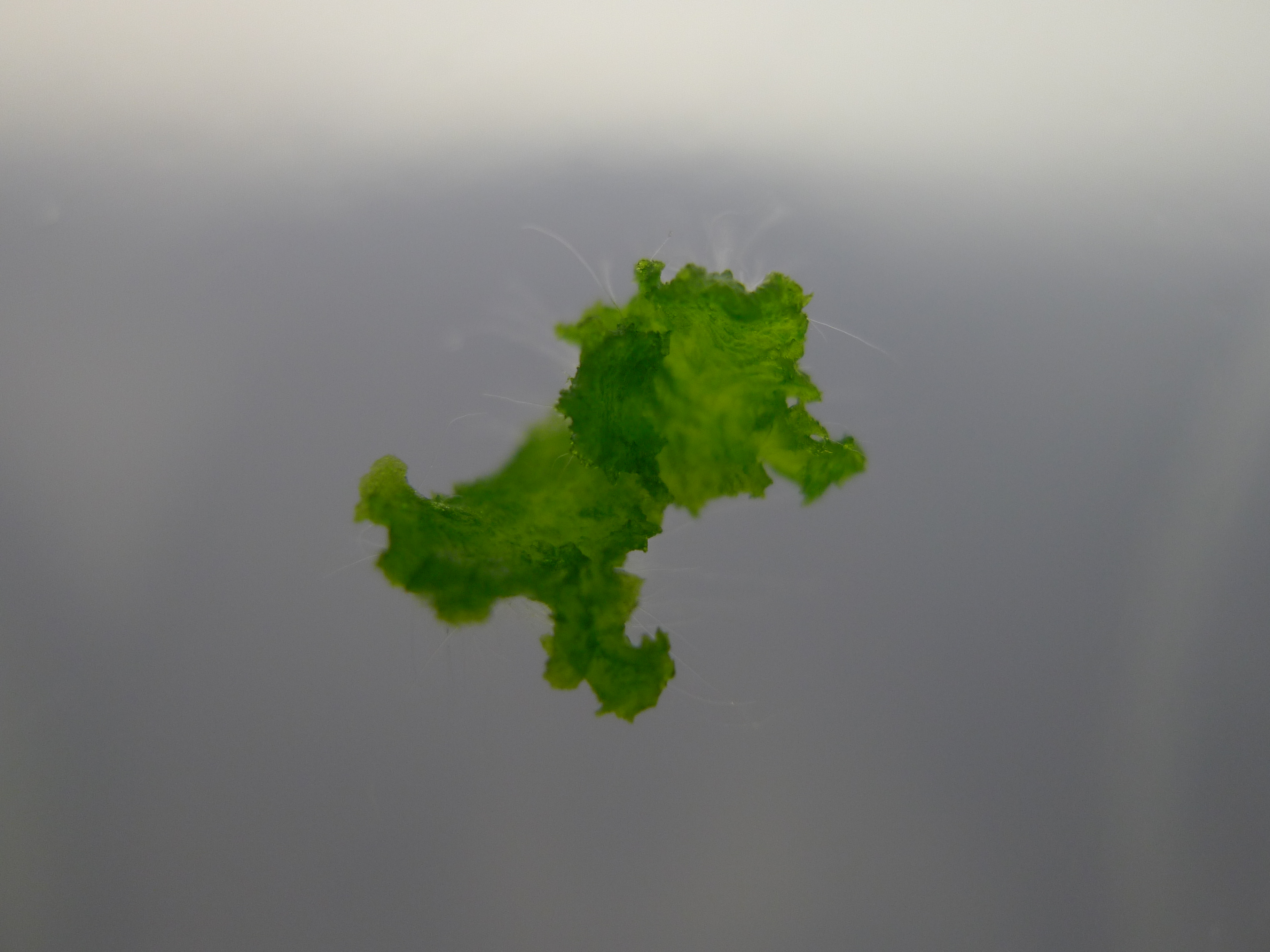

Supplement: Supplementary file 13 — Source data Fig. 5 [file 44318_2024_181_MOESM13_ESM.zip › Figure 5/5C/Mpcyp73a1-2.JPG]

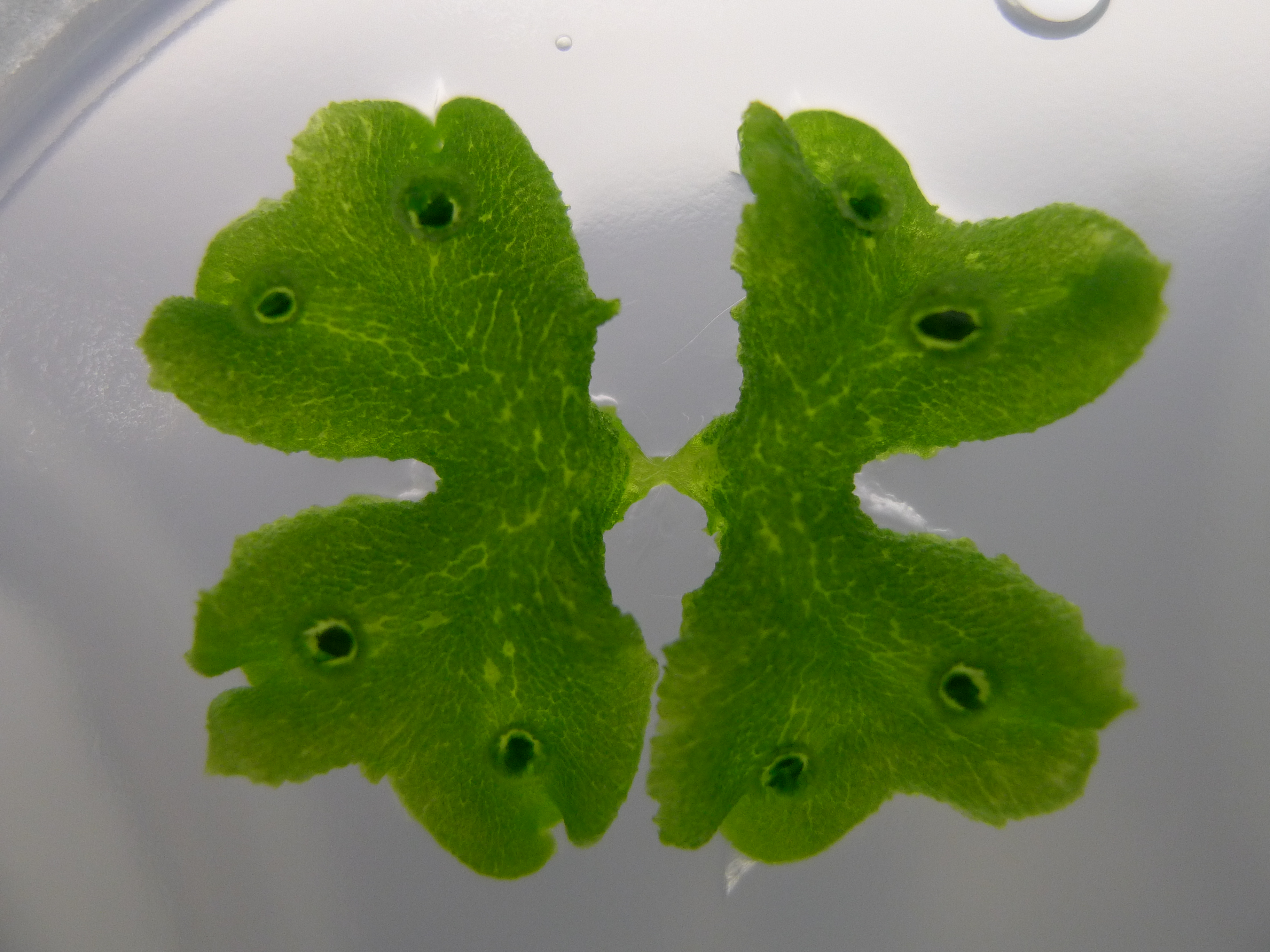

Supplement: Supplementary file 13 — Source data Fig. 5 [file 44318_2024_181_MOESM13_ESM.zip › Figure 5/5C/Wild_type.JPG]

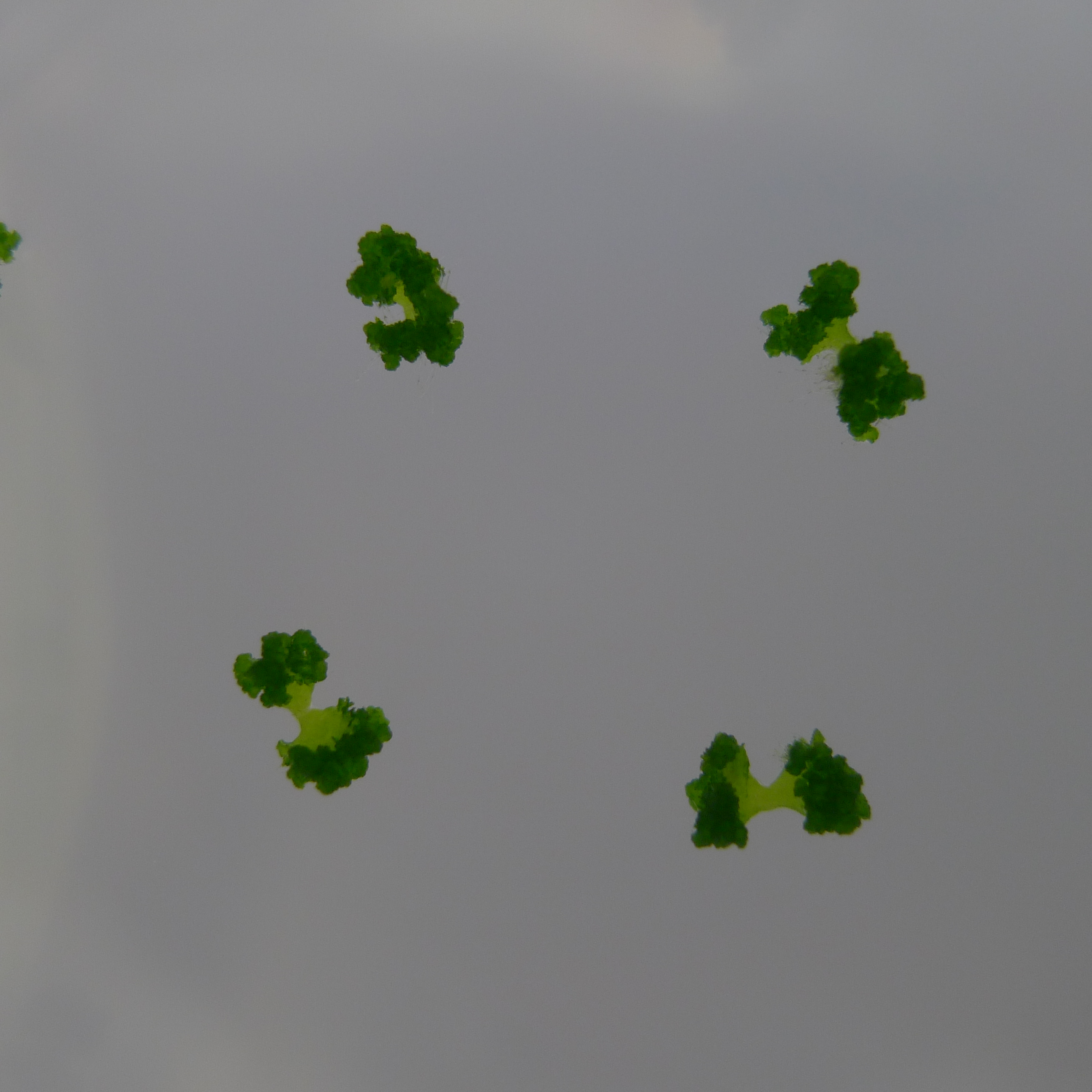

Supplement: Supplementary file 14 — Source data Fig. 6 [file 44318_2024_181_MOESM14_ESM.zip › Figure 6/6A/Marchantia_polymorpha_PA.JPG]

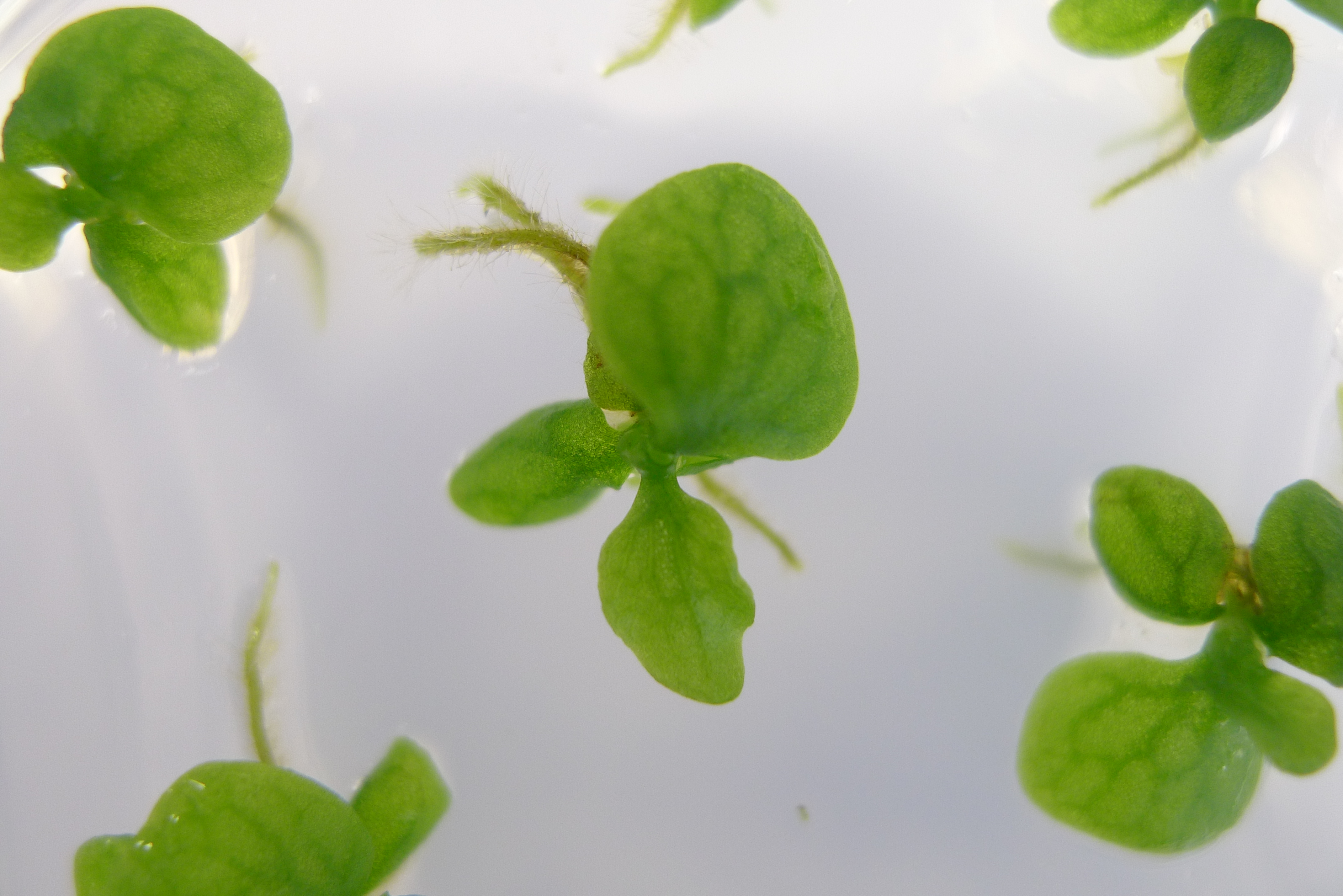

Supplement: Supplementary file 14 — Source data Fig. 6 [file 44318_2024_181_MOESM14_ESM.zip › Figure 6/6A/Ceratopteris_richardii_mock.JPG]

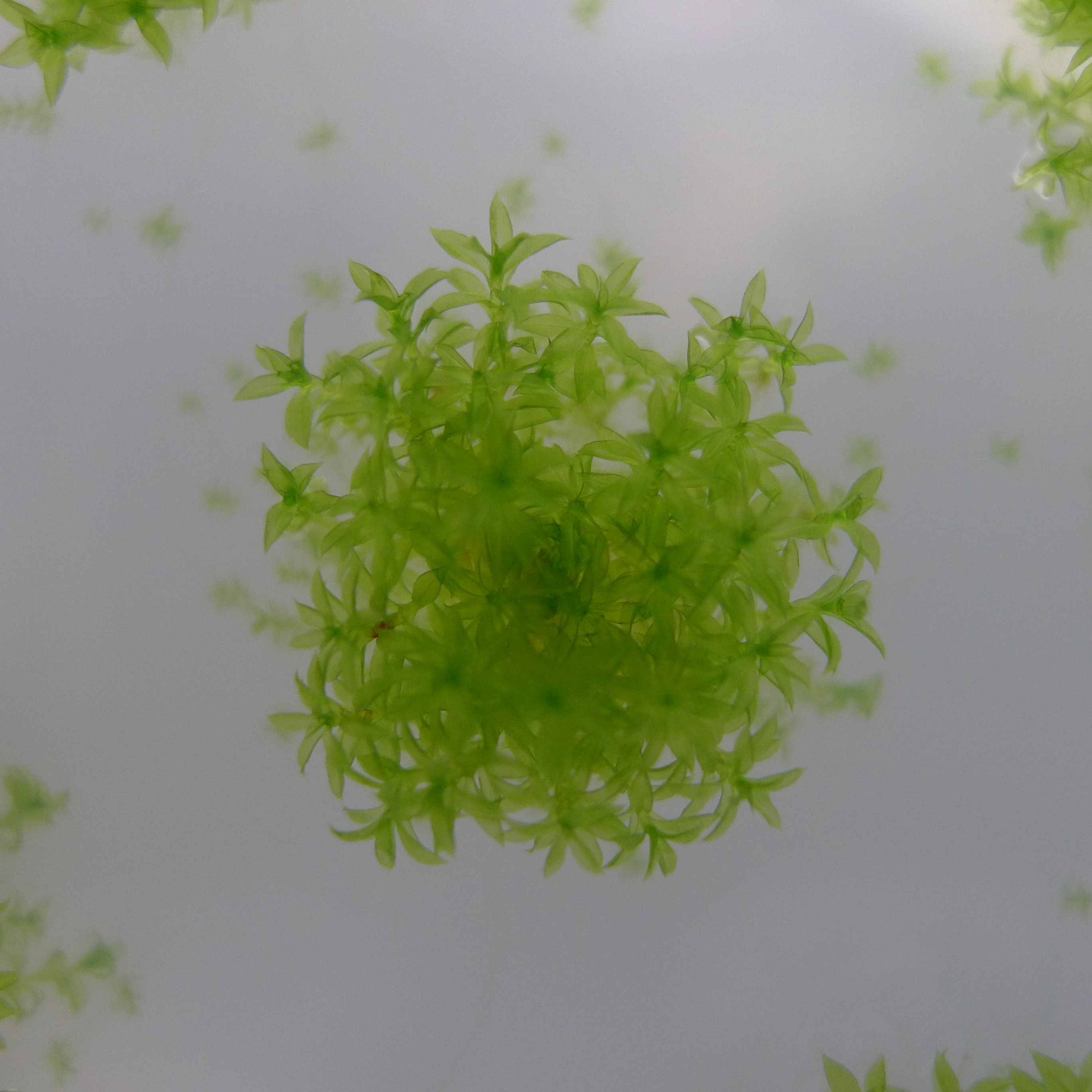

Supplement: Supplementary file 14 — Source data Fig. 6 [file 44318_2024_181_MOESM14_ESM.zip › Figure 6/6A/Physcomitrium_patens_mock.JPG]

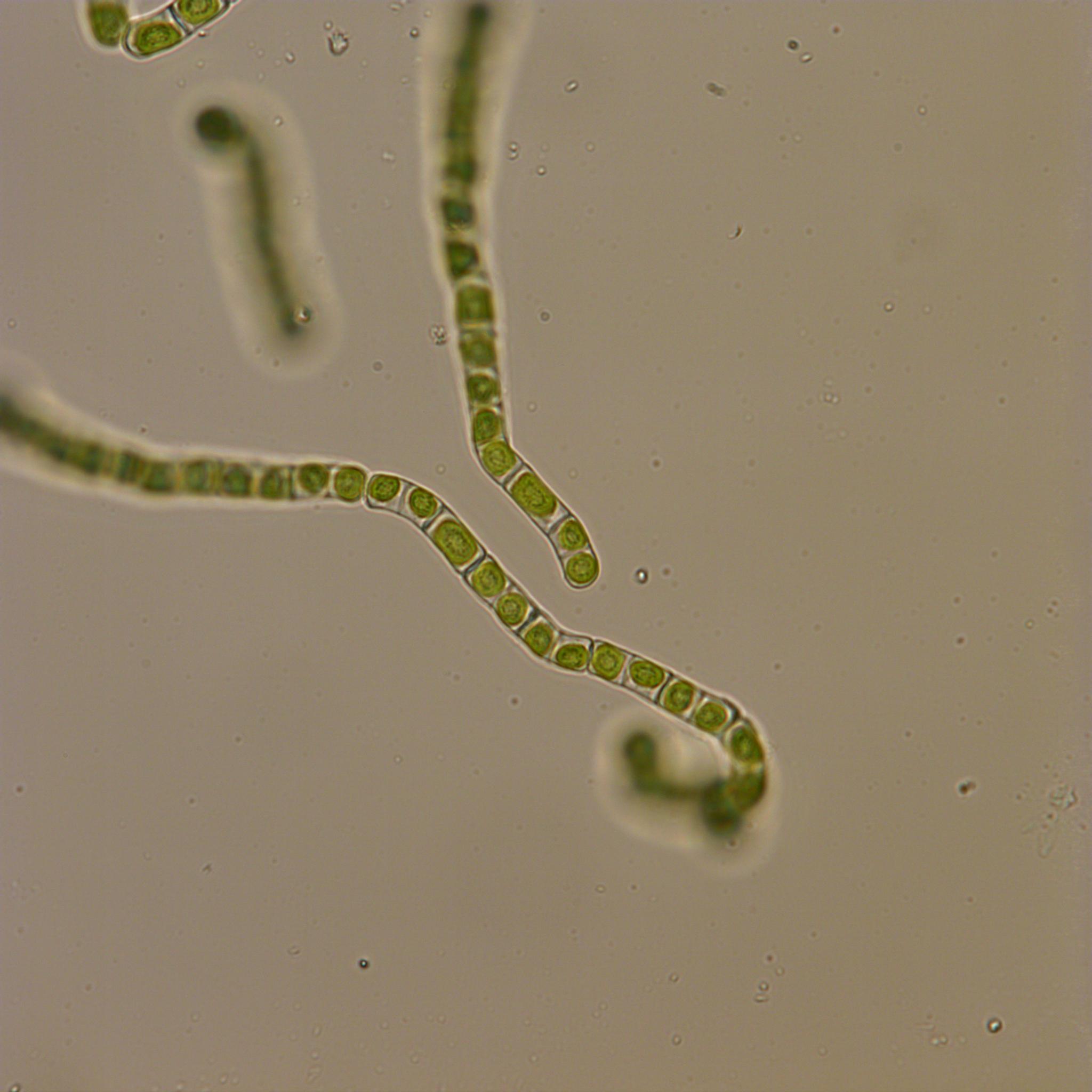

Supplement: Supplementary file 14 — Source data Fig. 6 [file 44318_2024_181_MOESM14_ESM.zip › Figure 6/6A/Klebsormidium_nitens_mock.jpg]

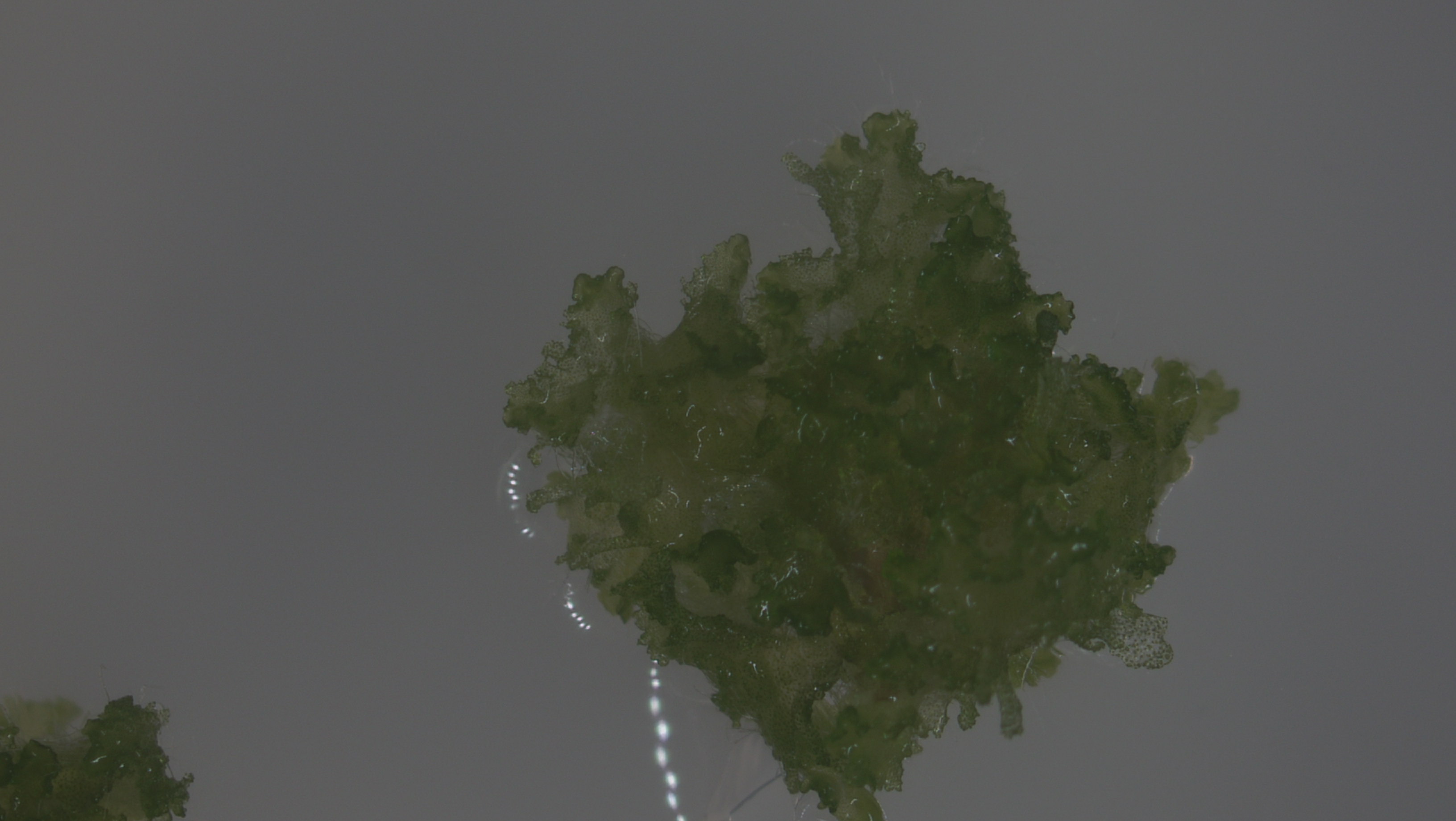

Supplement: Supplementary file 14 — Source data Fig. 6 [file 44318_2024_181_MOESM14_ESM.zip › Figure 6/6A/Anthoceros_agrestis_PA.JPG]

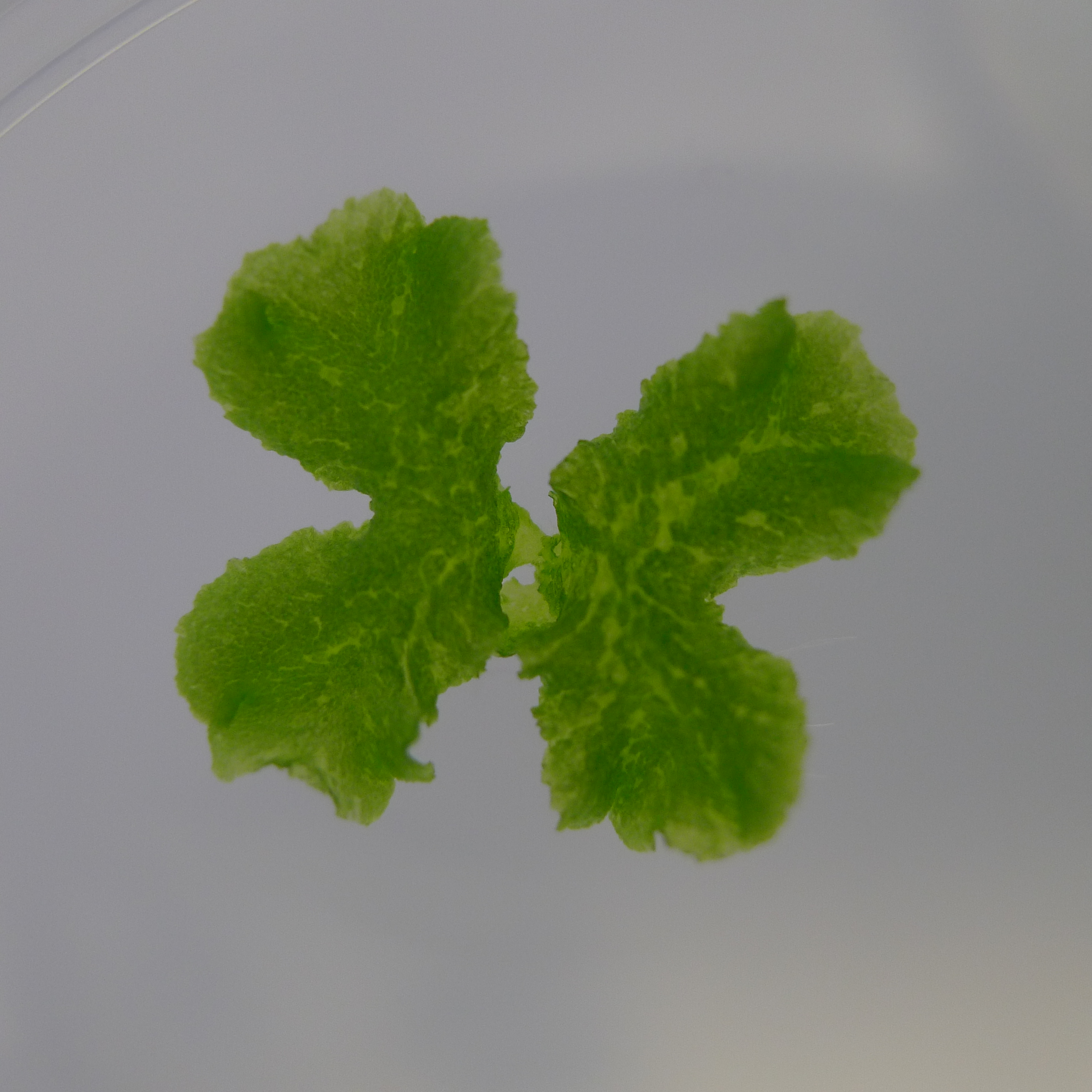

Supplement: Supplementary file 14 — Source data Fig. 6 [file 44318_2024_181_MOESM14_ESM.zip › Figure 6/6A/Marchantia_polymorpha_mock.JPG]

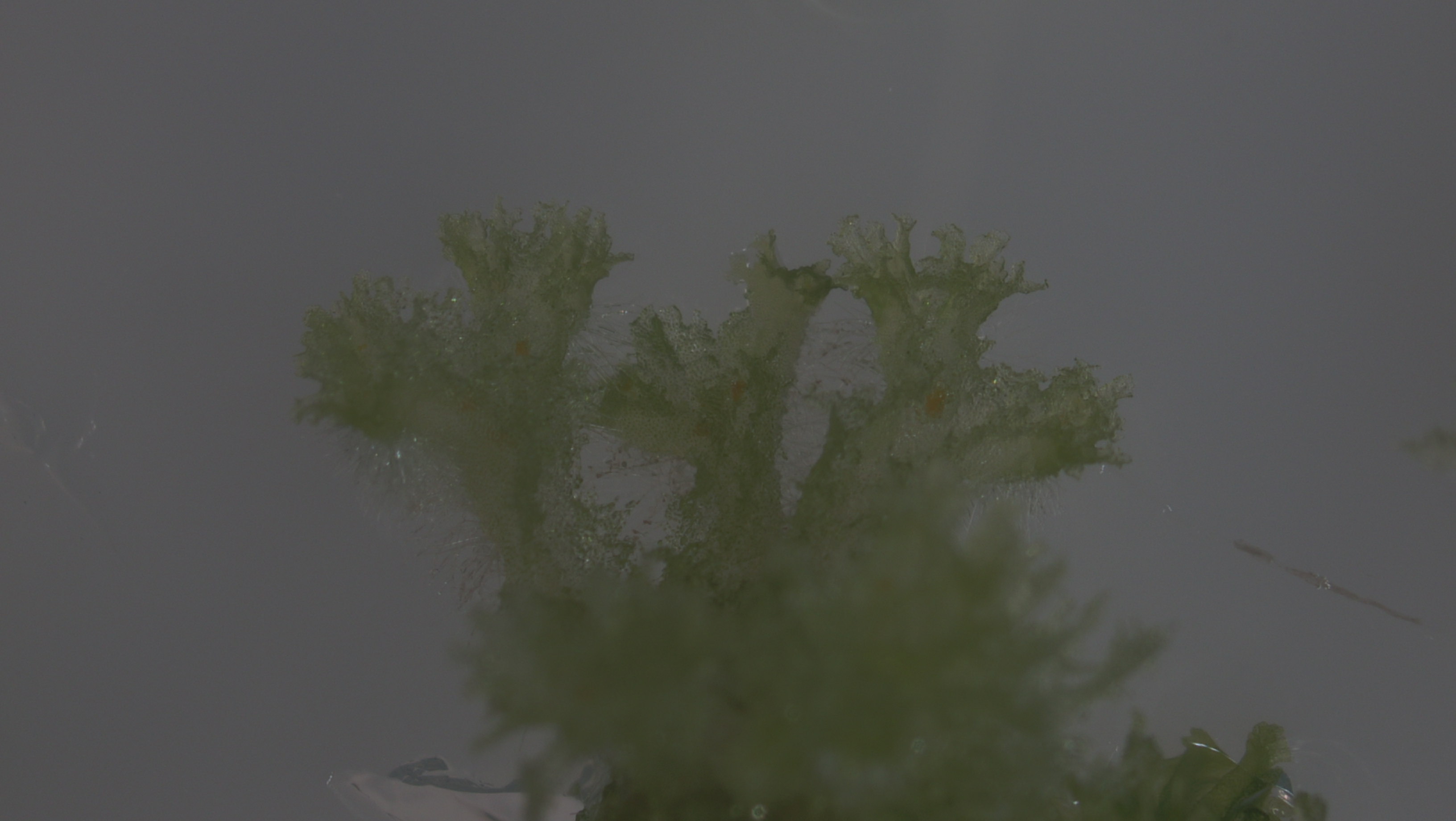

Supplement: Supplementary file 14 — Source data Fig. 6 [file 44318_2024_181_MOESM14_ESM.zip › Figure 6/6A/Anthoceros_agrestis_mock.JPG]

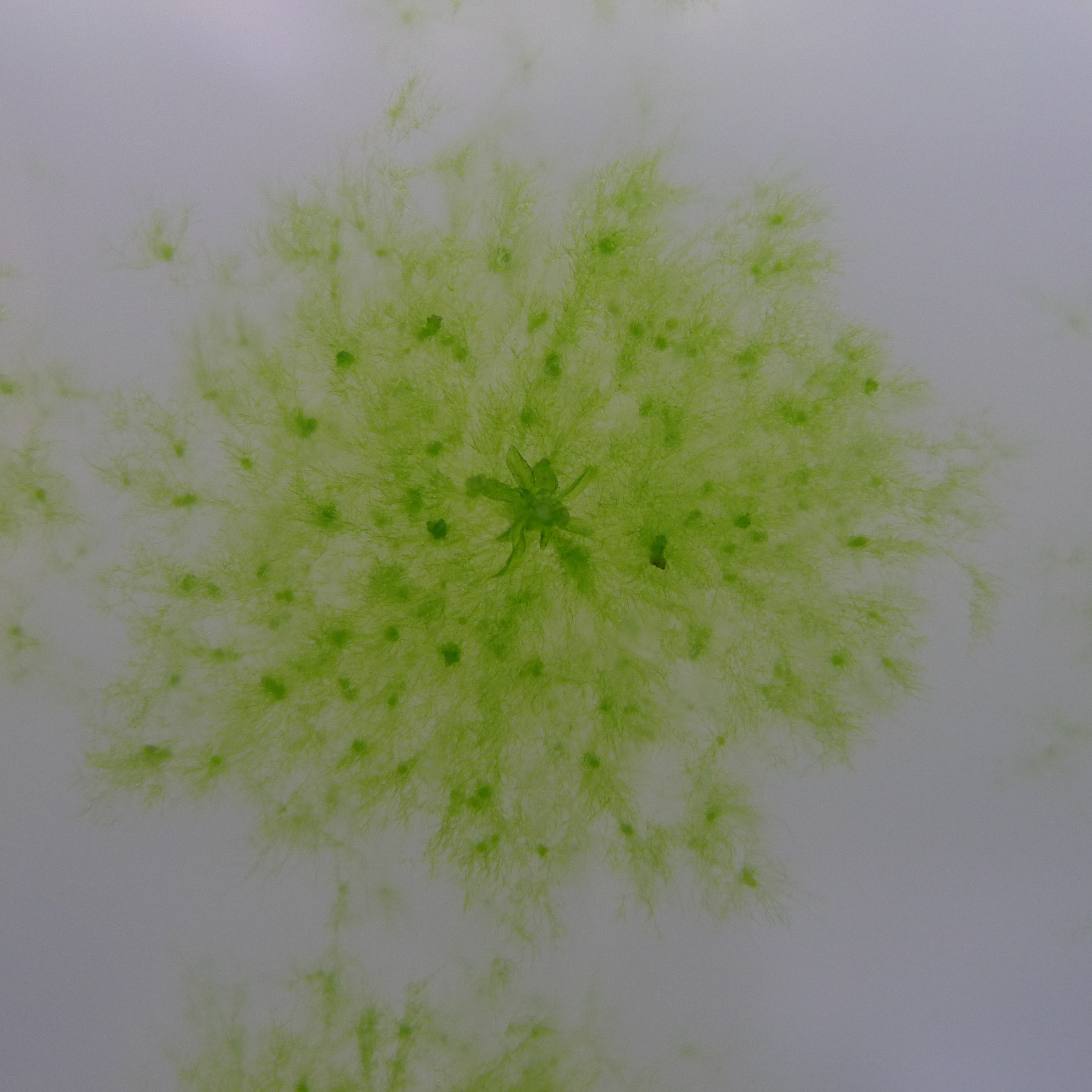

Supplement: Supplementary file 14 — Source data Fig. 6 [file 44318_2024_181_MOESM14_ESM.zip › Figure 6/6A/Physcomitrium_patens_PA.JPG]

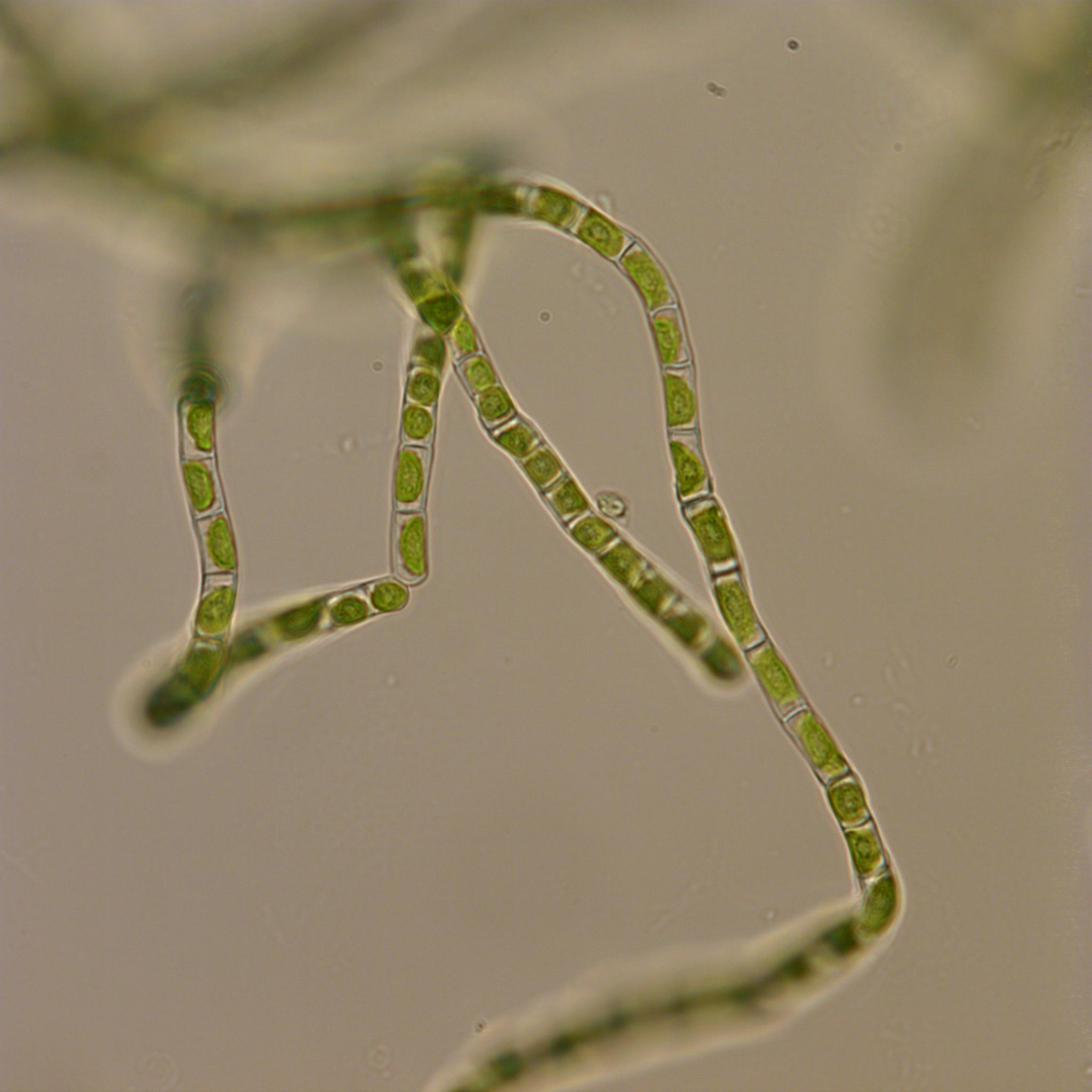

Supplement: Supplementary file 14 — Source data Fig. 6 [file 44318_2024_181_MOESM14_ESM.zip › Figure 6/6A/Klebsormidium_nitens_PA.jpg]

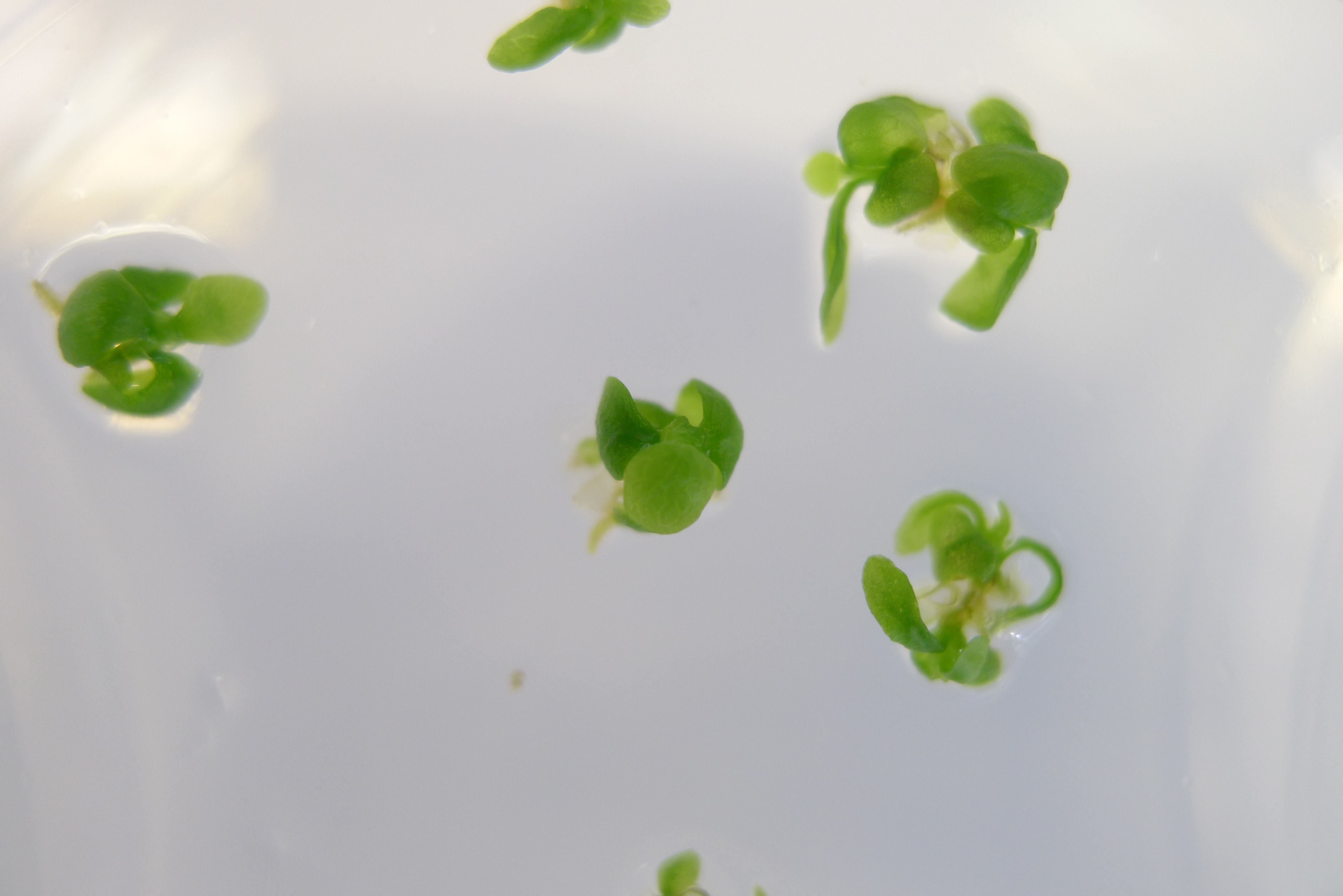

Supplement: Supplementary file 14 — Source data Fig. 6 [file 44318_2024_181_MOESM14_ESM.zip › Figure 6/6A/Ceratopteris_richardii_PA.JPG]
