# Supplementary material for: An ancient role for CYP73 monooxygenases in phenylpropanoid biosynthesis and embryophyte development
Source: EMBO J. 2024 Aug 1;43(18):13. doi: 10.1038/s44318-024-00181-7 (PMC11405693; doi:10.1038/s44318-024-00181-7)
Supplement: Supplementary file 15 — Expanded View Figures [file 44318_2024_181_MOESM15_ESM.pdf]

Expanded View Figures

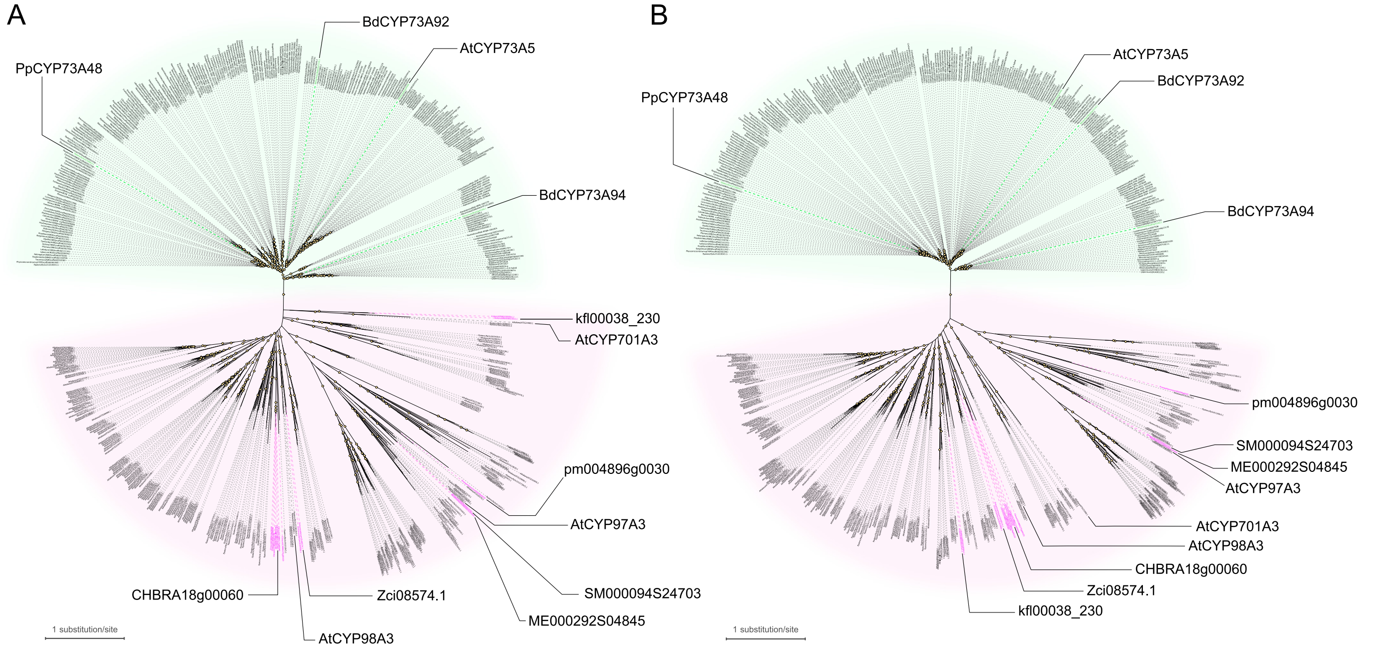

Figure EV1. Phylogenetic analysis of CYP73 and clan 71 CYP sequences.

Maximum-likelihood trees of 275 CYP73 sequences and 271 additional outgroup sequences derived from *A. thaliana*, *P. patens* and charophytes (AtCYP73A5 tBLASTn bit-score >100). (**A**) Unrooted ML nucleotide tree (IQ-TREE2, SYM + I + R10). (**B**) Unrooted ML amino acid tree (IQ-TREE2, Q.insect+I + R8). Relevant CYP73 homologs, *A. thaliana* clan 71 CYP members and charophyte sequences corresponding to BLASTp best hits (see Appendix Tab. S1) are shown. Ultrafast bootstrap support values equal or superior to 80 are annotated on branch as yellow dots. Trees are drawn to scale; scale bars represent the number of substitutions per site.

© The Author(s)

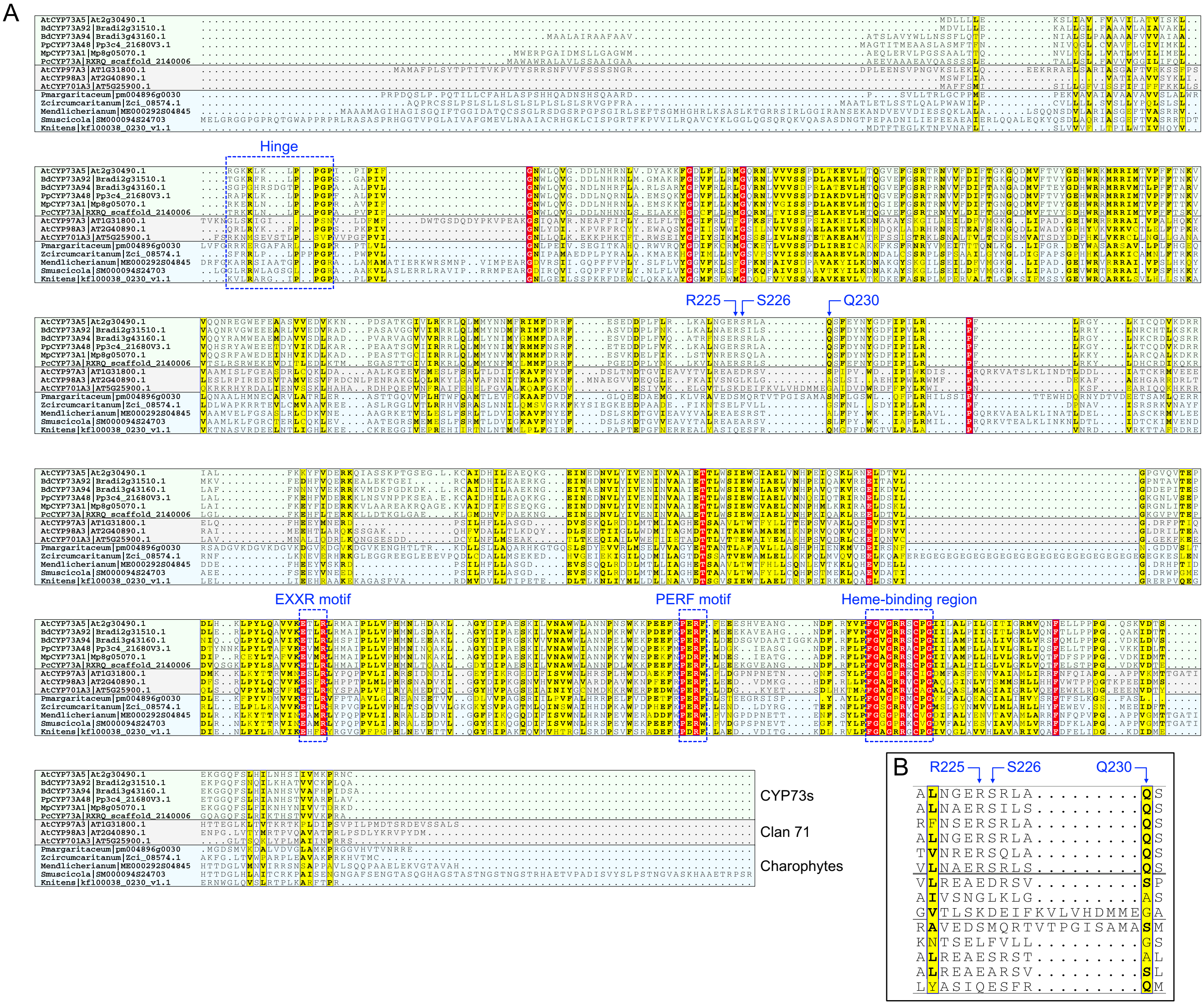

Figure EV2. Multiple sequence alignment of CYP73 proteins with clan 71 CYP members.

(**A**) Full-length protein sequences of biochemically characterized CYP73 proteins from both tracheophytes (AtCYP73A5, BdCYP73A92, BdCYP73A94) and bryophytes (PpCYP73A48, MpCYP73A1, PcCYP73A) were aligned with *A. thaliana* clan 71 CYP members (AtCYP97A3, AtCYP98A3, AtCYP701A3). Protein sequence corresponding to BLAST best hits in the charophytes *P. margaritaceum*, *Z. circumcaritanum*, *M. endlicherianum*, and *K. nitens* (Fig. 1B) were included in the alignment. These charophyte proteins were found closely associated to A. *thaliana* clan 71 CYP members (Appendix Tab. S1, Fig. EV1). Alignment was performed with MUSCLE and visualized using ESPript 3.0. Positions that are identical are highlighted with a red background; positions with >70% similarity are highlighted with a yellow background. Cytochrome P450 conserved regions are highlighted. (**B**) Focus on the protein region encompassing the three residues critical for *t*-cinnamic acid binding in CYP73 active site.

 © The Author(s)
